# Different Levels of Catabolite Repression Optimize Growth in Stable and Variable Environments

**DOI:** 10.1371/journal.pbio.1001764

**Published:** 2014-01-14

**Authors:** Aaron M. New, Bram Cerulus, Sander K. Govers, Gemma Perez-Samper, Bo Zhu, Sarah Boogmans, Joao B. Xavier, Kevin J. Verstrepen

**Affiliations:** 1 VIB Laboratory of Systems Biology, Leuven, Belgium; 2 CMPG Laboratory of Genetics and Genomics, KU Leuven, Leuven, Belgium; 3 Program in Computational Biology, Memorial Sloan Kettering Cancer Center, New York, New York, United States; University of British Columbia, Canada

## Abstract

Organisms respond to environmental changes by adapting the expression of key genes. However, such transcriptional reprogramming requires time and energy, and may also leave the organism ill-adapted when the original environment returns. Here, we study the dynamics of transcriptional reprogramming and fitness in the model eukaryote *Saccharomyces cerevisiae* in response to changing carbon environments. Population and single-cell analyses reveal that some wild yeast strains rapidly and uniformly adapt gene expression and growth to changing carbon sources, whereas other strains respond more slowly, resulting in long periods of slow growth (the so-called “lag phase”) and large differences between individual cells within the population. We exploit this natural heterogeneity to evolve a set of mutants that demonstrate how the frequency and duration of changes in carbon source can favor different carbon catabolite repression strategies. At one end of this spectrum are “specialist” strategies that display high rates of growth in stable environments, with more stringent catabolite repression and slower transcriptional reprogramming. The other mutants display less stringent catabolite repression, resulting in leaky expression of genes that are not required for growth in glucose. This “generalist” strategy reduces fitness in glucose, but allows faster transcriptional reprogramming and shorter lag phases when the cells need to shift to alternative carbon sources. Whole-genome sequencing of these mutants reveals that mutations in key regulatory genes such as *HXK2* and *STD1* adjust the regulation and transcriptional noise of metabolic genes, with some mutations leading to alternative gene regulatory strategies that allow “stochastic sensing” of the environment. Together, our study unmasks how variable and stable environments favor distinct strategies of transcriptional reprogramming and growth.

## Abstract 2

This study uses experimentally evolved brewer's yeasts to explore the costs and benefits of different nutrient-switching strategies when energy sources vary or remain constant.

## Author Summary

When microbes grow in a mixture of different nutrients, they repress the metabolism of nonpreferred nutrients such as complex carbohydrates until preferred nutrients, like glucose, are depleted. While this “catabolite repression” allows cells to use the most efficient nutrients first, it also comes at a cost because the switch to nonpreferred nutrients requires the de-repression of specific genes, and during this transition cells must temporarily stop dividing. Naively, one might expect that cells would activate the genes needed to resume growth in the new environment as quickly as possible. However, we find that the length of the growth lag that occurs when yeast cells are switched from the preferred carbon source glucose to alternative nutrients like maltose, galactose, or ethanol differs between wild yeast strains. By repeatedly alternating a slow-switching strain between glucose and maltose, we obtained mutants that show shortened lag phases. Although these variants can switch rapidly between carbon sources, they show reduced growth rates in environments where glucose is available continuously. Further analysis revealed that mutations in genes like *HXK2* cause variations in the degree of catabolite repression, with some mutants showing leaky or stochastic maltose gene expression. Together, these results reveal how different gene regulation strategies can affect fitness in variable or stable environments.

## Introduction

A stable environment generally favors organisms that are well-adapted to that specific niche [1]–[3]. However, in many cases, adaptation to one environment comes at costs to fitness in alternative niches [1],[4]–[9]. Aside from the fitness tradeoffs, adaptation through mutation is relatively slow. Thus to deal with certain recurring environmental changes, many organisms have evolved the capacity to change gene expression in response to the environment, reducing the need for genetic adaptation.

Microbial nutrient uptake and metabolism is a prime example of how organisms use transcriptional regulation to optimize fitness in variable environments. Because the expression of nonnecessary metabolic routes and genes is costly [3],[10],[11], microbes often use catabolite repression mechanisms to preferentially consume nutrients that afford high growth rates. This way, nonpreferred nutrient genes are only expressed when other, more preferred nutrients have been depleted. The sensing and signaling cascades required for carbon catabolite repression in the yeast *Saccharomyces cerevisiae* are particularly well-studied and serve as a model for similar systems in higher eukaryotes [12]–[15]. Glucose acts as a primary signal, triggering a regulatory cascade that results in repression of the consumption of alternative carbon sources, such as maltose, galactose, or ethanol. The main mechanism by which glucose regulates transcription is via the Ras/protein kinase A (PKA) signal transduction pathway. Other effectors include Snf1, the yeast homologue of mammalian AMP-activated PK, and Rgt1. Both of these proteins effect catabolite repression by triggering the transcriptional rewiring of a small subset of genes, many of which are directly involved in the uptake and metabolism of alternative carbon sources [12]–[14],[16].

Like other gene regulation programs, catabolite repression reduces the fitness cost associated with unnecessary gene expression while preserving the possibility of growth in environments with different nutrients. However, the ability to adapt to changing environments appears to be intrinsically opposed to obtaining maximal fitness in stable environments [6],[17]–[20]. This is partly because maintaining a regulatory mechanism requires energy and provides little benefit in stable environments [1],[21]–[25]. Moreover, in variable environments, transcriptional reprogramming also requires energy and time. This is clearly manifested when microbes switch from growth on one carbon source to another. During such a switch, cells show temporarily reduced growth rates (a so-called “lag phase”) because transcription is not yet adapted to the new environment [1],[26],[27]. As a consequence, an organism's fitness in a given environment depends not only on the maximal reproductive rates in that particular niche, but also on the speed with which gene expression can adapt to the new conditions [1],[8],[27]–[29].

Because maintaining and operating an environmental sensing system is not always beneficial, it has been suggested that some environments might favor simpler strategies. For example, certain variable and unpredictable environments might stimulate strategies where a clonal population uses random (stochastic) switching between phenotypically different states. Such so-called “bet-hedging” strategies can generate phenotypic diversity independently of present environmental conditions. If this diversity correlates with the frequency of environmental uncertainty, this will be an “evolutionarily stable” strategy that ensures that some portion of the population is always adapted to future conditions. This strategy reduces the duration of the lag phase and also avoids fitness costs associated with maintaining an environmental sensor [1],[19],[30]–[33]. Other studies have proposed that microbes can evolve mixed gene regulation strategies that combine sensing with stochastic switching. Such “stochastic sensing” strategies use clues about future environmental changes to induce anticipatory transcriptional changes in a portion of individuals within the population [1],[2],[29],[34]–[38].

Whereas the molecular cascades underlying gene regulation have been extensively studied, the natural diversity and fitness costs and benefits of different gene regulation strategies have received less attention. This is in part because accurately measuring fitness across different environments is challenging. Because of the exponential nature of population growth, the long-term expected fitness of an organism is determined by its geometric mean growth rate (GMR) across every environment it encounters, weighted by the frequency and duration with which these environments occur [1],[4]–[6],[27],[39],[40]. Hence, even short periods of low fitness may have a significant effect on the long-term performance of an organism. “Generalist” strategies affording similar but more modest fitness levels across different environments can therefore result in a higher geometric mean fitness, even if maximal fitness in certain preferred environments is reduced [2],[41]–[43].

In this study, we use a combination of population- and single-cell-level measurements of the model eukaryote *S. cerevisiae* to explore how different environments shape fitness and transcriptional regulatory strategies. More specifically, we measure fitness as cells grow in environments with a stable supply of glucose compared to environments where cells need to transition from one carbon source to another. We find that different natural yeast strains show large differences in the speed with which they are able to adapt gene expression and growth to changes in carbon sources. Using experimental evolution, we demonstrate that growing a strain that shows slow transcriptional reprogramming in a variable environment frequently results in mutations in key regulatory genes such as *HXK2*. These mutations give rise to phenotypic “generalists” that thrive well in variable environments, with short lag phases, less stringent catabolite repression, and faster transcriptional reprogramming—at the expense of maximal growth rates (MaxRs) in a stable glucose environment. Many of these generalist isolates implement a transcriptional regulatory strategy mediated by “stochastic sensing” of alternative carbon sources, allowing cultures to maintain consistent fitness across different environments. Alternatively, the same selection regime can favor specialist mutants of an opposite character, which display tight catabolite repression and slow adaptation to new environments (long lag phases), but higher growth rates in stable glucose conditions. An experimentally validated mathematical model reveals how alternative regimes of variable carbon environments will favor one carbon catabolite repression strategy over another. Together, our results reveal that the speed with which genes are induced and repressed in response to environmental signals is a highly variable and evolvable trait. Our study moreover illustrates how distinct strategies of transcriptional reprogramming shape fitness in constant or variable environments.

## Results

### The Duration of the Lag Phase During Adaptation to Different Carbon Sources Differs Widely Among Yeast Strains

**Figure 1:**
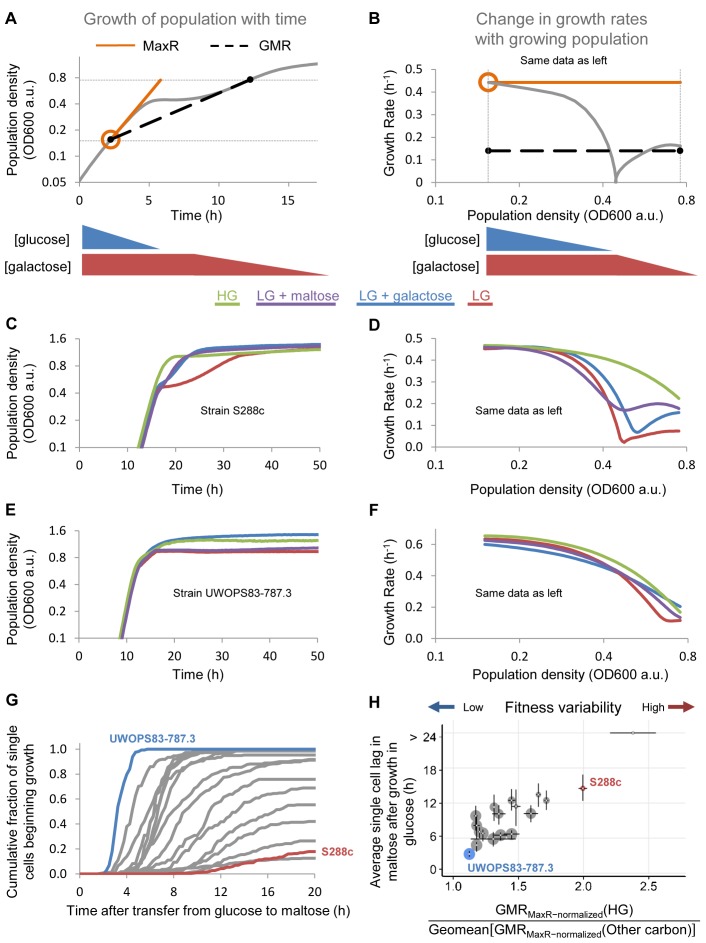
Yeast strains show large differences in the duration of the lag phase. (A) Example of a growth curve showing the biphasic growth associated with the switch from one carbon source to another (diauxic shift) of a strain (YS4) growing in the presence of LG supplemented with galactose. The figure shows a marked decrease in growth rate (lag phase) during the switch from glucose to maltose. MaxR is the maximal growth rate (or maximal fitness) attained at the beginning of the experiment when glucose levels are high, and correspond closely with growth rate measurements made of cells growing in very dilute conditions (not shown). GMR is a measure of average fitness throughout the experiment, calculated as the average growth rate between two preset cell densities that represent the beginning of measurable growth and the onset of stationary phase (see Materials and Methods for details). (B) Same data as panel (A), where the instantaneous growth rates of the culture are plotted as a function of population size. This representation shows more clearly how growth decelerates during the lag phase, leading to the often large difference between MaxR and GMR (black dotted line). (C and D) Growth pattern of a reference strain (S288c) with a pronounced lag phase growing either HG conditions (3% glucose, green) or 0.5% glucose, either alone (red) or supplemented with galactose (blue), maltose (purple). The growth rate in 3% glucose is relatively stable, whereas growth rates in the other media are more variable, with a temporary decrease typical of the lag phase when cells shift their metabolism from glucose to another carbon source. (E and F) Similar to (B) and (C) but with a strain UWOPS83-787.3 that shows almost equal fitness in different media. Note that the lag phase is barely detectable, and that growth only slows down at the end of the experiment, probably because of the depletion of nutrients and the accumulation of ethanol and other toxic metabolites. (G) Live-cell microscopy of yeast populations shifting between glucose and other carbon sources allows measurement of the lag phase of individual cells. Each curve represents the cumulative distribution histogram of single-cell lag phases of 1 of 18 different yeast strains. Each trace represents the fraction of a population of one given strain that has escaped the lag phase after a transfer from glucose to maltose as measured by budding events (Materials and Methods). The histograms reveal large differences in lag duration between strains, as well as variation in lag duration between individual cells within populations. One strain was omitted from this analysis because fewer than 1 in 150 cells resumed growth after transition to maltose. (H) Correlation between the average single-cell lags from (1 g) and population-level fitness variability (i.e., the variability of the GMR across different growth media). The vertical axis shows the average duration of a strain's lag phase (as measured by single-cell live microscopy), and error bars on this axis correspond to the lower and upper quartiles. The size of each data point is proportional to the fraction of cells that were observed to resume growth after transition to maltose. The horizontal axis represents the ratio of a strain's fitness in media requiring diauxic shift (LG, LG + galactose, and LG + maltose), relative to its fitness in stable HG conditions. Error bars on this axis are the standard deviations of 1,000 repeated calculations of the statistic obtained by random sampling of one biological replicate from each condition (*n* = 2–6 per strain in each condition). See also main text, Dataset S1, and Figure S1.

To investigate how the lag phase can influence fitness, we compared the growth behavior of 18 different *S. cerevisiae* strains in stable and variable environments. These included two commonly used laboratory strains (S288c and SK1) as well as 16 genetically diverse strains described by Liti et al. (2009). We used bulk population-level growth measurements in an automated plate reader (see Materials and Methods) to measure fitness in four different environments, including one stable condition with abundant glucose and three variable environments where populations gradually run out of glucose and thus need to switch to a different carbon source to continue growth. To obtain a stable “high glucose” (HG) condition, we supplemented the growth medium with 3% glucose, a condition that allowed relatively constant growth rates until cells entered stationary phase. At the other extreme we supplemented growth media with only 0.5% glucose (low glucose, LG), a condition that allows cells to first grow quickly by fermenting glucose, and then reprogram their metabolic genes to switch to respiratory growth on the ethanol accumulated during the fermentation phase (Figure 1 and Figure S1). Two other variable conditions included supplementation of LG with either maltose or galactose, two nonpreferred fermentable carbon sources whose metabolism is repressed in the presence of glucose (Figure 1 and Figure S1).

As expected, many yeast strains showed a clear lag phase when grown in media that induce an adaptation to new carbon sources (called a “diauxic shift”) (Figure 1 and Figure S1). The shift can be detected by three characteristic changes in growth rate: a deceleration in growth speed as glucose is depleted, a brief phase where the growth rate reaches a (local) minimum, and subsequent re-acceleration to adapted growth on the alternative carbon source (Figure 1A,B and Figure S1A). The lag phase was especially pronounced in media with LG alone, and more subtle or in some cases completely absent in LG + galactose and LG + maltose. However, even when the growth deceleration during the diauxic shift is very pronounced, it is not trivial to accurately quantify the lag phase because populations rarely arrest growth completely, and because the start and end of the phase cannot be clearly defined. Furthermore, the deceleration and local minimal phases appeared to be affected by the presence of an alternative carbon source (Figure S1A), and this effect was highly variable between strain backgrounds. For example, S288c growing in LG + maltose initially decelerates faster than the LG condition, but subsequently shows significantly higher growth rates across the rest of the experiment. By contrast, the LG + galactose sample has a higher rate of growth throughout the deceleration phase but decelerates later on in the curve as the culture adapts fully to galactose consumption (Figure 1C,D and Figure S1A). Moreover, these differences are strain-dependent. Strain Y55, for example, shows a more pronounced decrease in growth speed during the diauxic shift in LG + galactose compared to LG alone (Figure S1A).

The results above show that it is difficult to quantify the population-level lag phase by simply measuring its duration. In order to quantify the rate with which strains are able to adapt to variable environments, we therefore use a simple metric that summarizes the overall growth speed (or fitness) of a population as it transitions through a diauxic shift. This GMR is the weighted geometric mean of growth rate values across the shift in carbon conditions and represents the average growth rate of the strain across a specific interval. We chose to calculate the GMR for the interval between O.D. 0.15 and O.D. 0.75, which comprise the complete shift from one carbon source to another (see Materials and Methods and Figure 1A,B). The GMR can approach but never exceed the MaxR achieved by the culture while it was growing on the preferred carbon source glucose. Hence, the longer that a population of cells grows in stable glucose conditions, the closer its GMR approaches the culture's MaxR. In mixed media, by contrast, the GMR can be considerably lower than MaxR because of the decline in growth speed as cultures transition to growth in the nonpreferred carbon sources. Therefore, the ratio of the GMR in stable versus variable conditions is a measure for how efficiently the cells can transition between the different conditions, which in turn largely depends on the severity of the lag phase (see also Materials and Methods).

Examining the GMR across growth in stable (HG) and variable (LG, LG + maltose and LG + galactose) conditions allowed us to quantify how the carbon environment affected the overall fitness of the different strains. As expected, for all strains, the highest GMR was found in stable (HG) conditions, whereas the LG condition resulted in the lowest GMR (Dataset S1). Remarkably, however, the difference between these bounds was highly variable: whereas some strains only showed a 10% reduction in GMR in LG conditions compared to HG conditions, others showed a 70% reduction in GMR. A similar although less pronounced variation between strains was also observed for LG + galactose and LG + maltose media (Figure S1B and Dataset S1).

Together, these results indicate that the diauxic shift from glucose to a less preferred carbon source leads to a wide array of growth behaviors, ranging from highly variable growth rates in different media to almost constant growth rates throughout the shift in carbon source. The decrease in fitness during diauxic shift depends both on the yeast strain and the carbon source. In general, the transition from glucose to ethanol (LG medium) induced the strongest decrease in growth rate (and thus also GMR), whereas the glucose to maltose transition (LG + maltose medium) caused the smallest reduction compared to stable HG conditions (Figure S1B).

### Sudden Carbon Source Shifts Can Elicit Long and Heterogeneous Lag Phases Across Single Cells

To obtain a more detailed picture of the decrease in fitness during the lag phase as cells transition from one carbon source to another, we turned to single-cell measurements. The cells were first grown in glucose, harvested, and then immediately transferred to maltose-containing media. The duration of the lag phases of individual cells after this sudden transfer to maltose was measured using automated time-lapse microscopy (Materials and Methods and Movies S1 and S2). We recorded the lag phases of individual cells as the time it took a cell to begin dividing after transfer from glucose to maltose-containing medium (i.e., the first appearance of a cell bud or the resumption of bud growth). The results of these experiments indicate that the 18 yeast strains showed a striking diversity in the average duration of the lag phase (Figure 1G). Interestingly, even within a population of a given strain, we often observed a large diversity in lag duration among different individual cells. Moreover, in some cases, not all cells seemed to survive the shift in carbon source. The degree of intraclonal variability in lag duration (measured by the standard deviation of single-cell lag phases of a population of cells of a given strain) and fraction of cells surviving within 20 h of recording was significantly related to the average lag phase, an observation that held true for all strains and mutants we examined in this study (Figure S1C–F).

Strikingly, the average duration (and thereby heterogeneity) of the single-cell lag phase of a given yeast strain was also highly anticorrelated with the fitness (GMR) measured in population-level experiments where strains often displayed pronounced lag phases, such as in LG and LG + galactose conditions. More generally, cells showing long lag phases as measured by single-cell microscopy also showed higher variation in fitness (standard deviation and coefficient of variation in GMR) between different growth media (HG, LG, LG + Gal, LG + Mal; see Figure 1H). Further statistical analysis using proportional hazard regression (see Text S1) confirmed this correlation (Dataset S1B).

Taken together, these analyses indicate that the efficiency with which populations are able to shift between carbon sources varies significantly between different strains, and is correlated with the average lag phase measured during sudden shifts in carbon source. Specifically, strains that show large differences in fitness between stable (HG) and variable (LG, LG + Maltose and LG + Galactose) environments also show long lag phases.

### Glucose Repression of the MAL Genes Leads to Long Lag Phases

The results from the previous section support that lag phases measured for single cells in sudden glucose to maltose shifts are correlated with lag phases measured by monitoring population-level growth in conditions where glucose is more gradually depleted (Figure 1H and Dataset S1). Interestingly, although sudden transitions from glucose to maltose media often led to long and heterogeneous lag phases, most cultures growing in LG + maltose mixed media displayed modest lag phases, suggesting that the yeast cells were able to maintain higher overall growth rates if the transition between glucose and maltose was more gradual. Exploring the molecular details of this shift from glucose to maltose proved to be an ideal model because only three genes are required for maltose consumption, making it a simple system to study. To grow on maltose, yeast cells need to express a maltose transporter (MalT), a maltase (MalS), and a regulator (MalR) that induces the genes in the presence of maltose via positive feedback regulation [44]. To characterize this phenomenon in further depth, we chose to work with the laboratory strain S288c because it is genetically tractable and displays a clear lag phase in glucose-to-maltose transitions.

It seemed likely that carbon catabolite-mediated repression of the *MAL* genes was a key factor contributing to the long lag phases in sudden glucose to maltose shifts. Using fusions of the *MAL* proteins with fluorescent reporters, we observed, as expected, that expression of *MAL* genes is repressed in the presence of glucose, and induced in the presence of maltose after glucose is depleted (Figure S2). Moreover, constitutive overexpression of the *MAL* genes resulted in a much shorter lag phase (logrank Chi^2^ = 591, *p*<1×10^−16^) that was comparable to some of the natural strains with short lag phases, suggesting that a long lag phase is due to the slow de-repression of *MAL* genes (Figure S2B). Taken together, these results suggest that the duration of the lag phase is determined at least in part by the gene expression state of cells upon transition to the new carbon source. In sudden shifts from glucose to maltose, lag phases appear to be longer and more heterogeneous due to the time required to activate the *MAL* genes, whereas in mixed LG + maltose medium, strains can prepare for maltose fermentation before glucose is completely depleted (Figure 1C,D and Figure S2).Therefore, the different lag phase behaviors are likely to be mediated by a variable extent of carbon catabolite repression across strains and conditions.

### Long, Heterogeneous Lag Phases Can Be Beneficial

**Figure 2:**
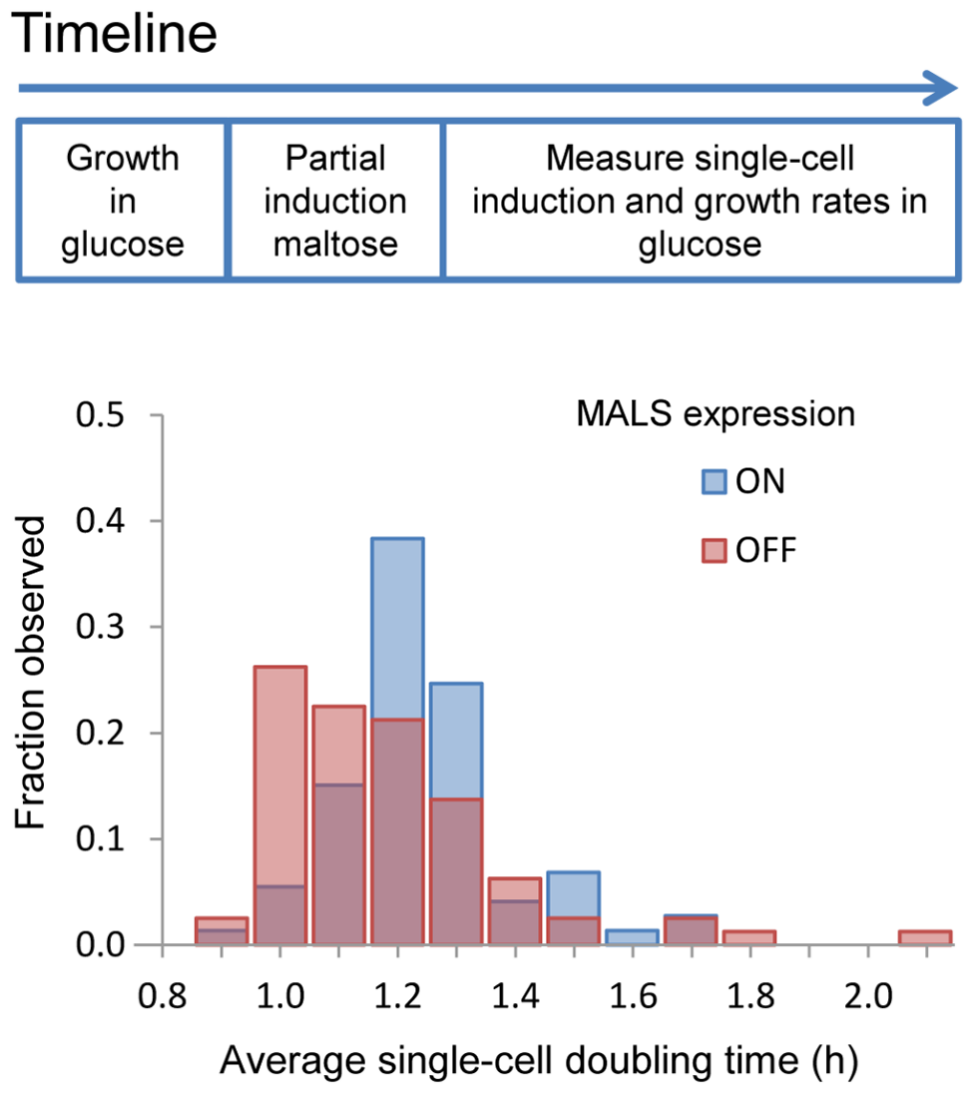
Rapid adaptation to a new carbon source can come at a fitness cost. A population of glucose-repressed MALS-YeCitrine reference strain (S288c) cells was grown in maltose until 50% of cells had escaped the lag phase and committed to growth on maltose, and then transferred back into glucose-containing media to measure the costs of commitment. Commitment to growth in maltose was measured as the cells' initial expression of the maltase-YeCitrine reporter. These cells were then tracked by time-lapse microscopy to allow growth rate measurements. The blue bars represent cells committed to growth on maltose, which grow at significantly longer doubling times after they were transferred back to glucose compared to sister cells that were still in the lag phase when they were transferred back to glucose (red bars). The results of a Mann–Whitney U test, reported in Figure S2E, show that these differences are robust for MAL “ON” cutoff values greater than background levels (*p*<0.01).

At first sight, it may seem suboptimal for strains to have long lag phases instead of rapidly adapting to a new environment. However, long lag phases might be adaptive under certain conditions [1]. For example, a long lag phase caused by carbon catabolite repression could potentially allow cells to more rapidly resume growth should preferred carbon sources like glucose return to the environment. To test this, we transferred a population of glucose-repressed cells to maltose, waited until half of the population had committed to maltose growth, as indicated by expression of a MalS-YeCitrine fluorescent reporter construct. We then transferred these cultures to glucose medium and measured the initial *MAL* fluorescence state of individual cells and subsequently tracked these cells' growth rates in glucose using time lapse microscopy (Figure 2 and Figure S2D,E). Compared to isogenic sister cells that had not yet escaped the lag phase, cells that already had activated their *MAL* genes showed lower growth rates in glucose for at least two cell divisions, showing that commitment to maltose growth came at a fitness cost when glucose reappeared. The large distribution in lag duration between isogenic cells in a population may therefore serve as a way to distribute the costs and benefits of commitment to nonpreferred nutrients across individuals within the population. Strains with tight catabolite repression, with longer and more heterogeneous distribution of single-cell lag times, appear to implement this strategy to a greater extent than strains with shorter and more homogeneous lag time distributions.

### The Lag Phase Is an Evolvable Trait

The above results indicate that the lag phase (and thus the speed of transcriptional reprogramming) has significant genetic determinants: there is a wide variation in lag duration between genetically distant yeast strains, and further we can engineer shorter lag phases with a reverse-genetics approach. Moreover, because it appeared that long lag phases could themselves be beneficial when glucose returns frequently to the environment (Figure 2), we reasoned that this trait should be subject to natural selection. To test this, we cycled the strain S288c between glucose and maltose to generate conditions of strong selective pressure. In the first leg of the cycle, to maintain selection on MaxR in preferred nutrients, cells were allowed ∼10 generations of exponential growth in stable glucose conditions. Then, in the second half of the cycle we selected for short lag phases by transferring these cultures into maltose-containing medium, allowing for ∼5 more generations of growth. In a first experiment, 12 populations expressed a constitutively transcribed YeCitrine marker to facilitate later analysis in competition experiments. After six cycles or ∼90 generations, 11 out of 12 evolving populations showed shorter lag phases when compared to the ancestral strain (Figure S3A and Movies S3, S4, S5).

**Figure 3:**
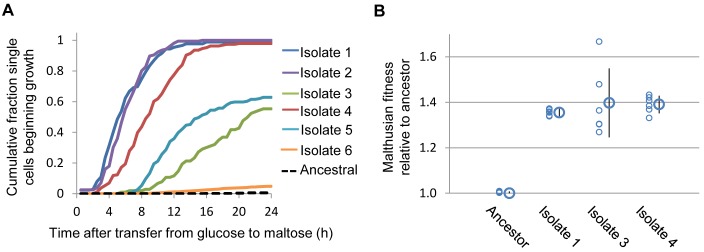
Experimental evolution in variable environments shapes the lag phase. Parallel cultures of a strain showing long lag phases were evolved in variable nutrient conditions, by transferring the cells back and forth between glucose and maltose medium. After 6–8 cycles, individual cells were isolated from the different cultures and their growth properties were analyzed (see main text for details). (A) Single-cell lag profiles from representative isolates from independently evolving populations, illustrating the diversity of glucose-to-maltose lag phase lengths. Note that a few isolates showed longer lag phases than the ancestral strain (Isolate 6, orange trace and Figure S3C). (B) The isolates are fitter than the ancestor in conditions mimicking the selection. Three of these isolates and the ancestor were directly competed against a reference strain in conditions mimicking the selection protocol (Materials and Methods). The large circles represent the average fitness relative to the ancestor of six biological replicates, and error bars represent standard deviations.

At the end of the experiment we isolated individual cells from each population. We found that clones isolated from different cultures varied widely in their single-cell lag profiles (Figure 3A), but that the profiles of individual isolates within each evolved population were similar (Dataset S2), suggesting that a single evolved phenotype had come to dominate each independently growing culture. The average single-cell lags of the isolated mutants range from as short as 5 h, comparable to the shortest lag phases observed in the collection of different strains reported in Figure 1, to clones with lags of longer duration than the ancestral strain (Dataset S2). The Malthusian fitness under conditions mimicking selection for each of three short-lagged isolates from independent populations was ∼1.35–1.4-fold higher than the ancestral strain (Figure 3B).

### Experimentally Evolved Strains Show Either Generalist or Specialist Population-Level Growth Properties

**Figure 4:**
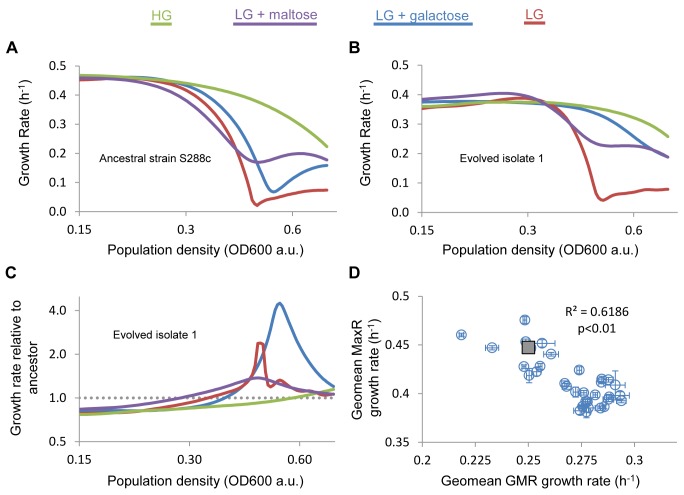
Fitness tradeoffs between rapid adaptation and MaxRs. Many evolved strains show reduced lag phases in variable environments (LG; LG + maltose or LG + galactose). (A) Growth pattern of the ancestral strain in either stable glucose conditions (HG or in media that require a shift from glucose to a less preferred carbon source (LG; LG + maltose or LG + galactose). (B) Similar analysis as panel (A) of Isolate 1 from Figure 3A. Note that the reduction in growth speed (lag phase) is much less pronounced than for the ancestral strain shown in panel (A). (C) Dividing the growth rates of the evolved clone by the ancestral reveals that the evolved clone grows more slowly than the ancestral strain, except during the lag phase, where the evolved isolate shows a much higher growth rate. This shorter lag phase is responsible for the increased GMR relative to the ancestral strain. (D) The MaxR and the GMRs are anticorrelated. Each point represents the geometric mean of the GMRs in all different conditions used in this study (LG, LG + galactose, LG + maltose, HG) versus the geometric mean of all MaxRs in the same conditions. Error bars represent standard deviations. The grey square represents the ancestral strain.

To investigate how reproducible this result was, and to unravel the underlying genetic and molecular mechanisms that allowed these strains to increase fitness, we repeated the evolution experiment, however this time using 12 populations of cells bearing *MALT*-*YeCitrine* and *MALS-mCherry* constructs to allow measurement of *MAL* gene expression. After eight cycles or ∼120 generations, all 12 populations in this experiment showed shorter lag phases (Figure S3B). We first carried out extensive growth rate analyses on 36 isolates (three clones from each of the 12 populations). Similarly to what we observed in the previous experiment, the majority of the mutants isolated after cycling populations between glucose and maltose medium showed shortened lag phases (Figure S3B,C). Using population-level growth measurements similar to those reported in Figure 1, we found that these evolved strains had smaller differences in fitness (GMR) between different growth environments than the parental strain, leading to overall higher fitness across the shift in carbon sources relative to the ancestral strain (Figure 4).

Interestingly, despite the fact that selection was only based on glucose-to-maltose cycles, the mutants also showed dramatic improvement in fitness in media containing LG alone or LG + galactose, conditions where the ancestral strain showed a pronounced lag phase (Figures 4 and S4). For example, several mutants no longer have any lag phase in galactose-containing media, maintaining steady rates of growth throughout the curve with no local growth rate minimum (Figures 4B and S4 and Dataset S2). This reduction in lag phase leads to a 1.2–1.4-fold higher fitness (GMR) during the diauxic shift. Likewise, several isolates have a GMR increase in LG conditions of up to 1.3-fold, an increase in fitness mediated by increased rates of growth throughout the deceleration, local minimum, and re-acceleration phases of the lag phase (Figure 4B,C and Figure S4). In LG + maltose conditions, where the ancestral strain showed a relatively limited lag phase (Figure 1C,D), we found that the evolved strains' fitness also showed a modest increase due to a further reduction of the lag phase.

Taken together, compared to the ancestor, the majority of evolved isolates developed a low degree of fitness variability (i.e., similar fitness levels in stable HG and variable conditions) and short lag durations that are similar to some of the strains measured in Figure 1 (Figure S4D and Datasets S1 and S2). Intriguingly, however, in addition to the isolates with increased fitness during transition between carbon sources, clones from a few populations showed an increase in lag duration compared to the parental strain (Figure 4D and Figure S4C,D). These isolates appear to have evolved higher MaxRs in stable glucose conditions at the cost of even more pronounced lag phases than the ancestral strain. When the average fitness (GMR) is plotted against the average MaxR, it becomes clear that isolates generally evolved following two different paths: the evolution of shorter lag phases and increased fitness during carbon transitions at the expense of MaxRs in stable glucose conditions, or the evolution of faster growth in stable conditions at the expense of longer lag phases in variable environments (Figure 4D and Dataset S2).

### Mutations in Key Regulators of Catabolite Repression Tune the Duration of the Lag Phase

To test which mutations might have given rise to short lag phases and increased fitness during carbon transitions in the evolved populations, we sequenced the genomes of four evolved isolates showing shorter lag phases. Three mutants of the shortest lag phase length carried mutations in the glucose sensor *HXK2*, a gene that encodes a protein with multiple genetically dissociable roles in glucose sensing. Specifically, in addition to phosphorylating glucose for entry into glycolysis, Hxk2p plays a signaling role in the *SNF1* and Ras/PKA pathway, and further can itself translocate into the nucleus to repress certain nonpreferred carbon catabolite genes [45]. High throughput studies have shown that clean deletions of this gene lead to reduced fitness in YPD media (a condition akin to our HG media, YPD contains 2% glucose) relative to the WT, and increased fitness in diauxic shift from 0.1% glucose to ethanol and glycerol [46]. Another study demonstrated that deletion of this gene leads to genome-wide disruption of transcription, with significant gene ontological (GO) enrichment for genes involved with respiration and nonpreferred carbon metabolism [47].

Another isolate (Isolate 3) showing longer lag phase duration carried a mutation in the *STD1* gene, encoding a protein that interacts with glucose sensors *Snf3* and *Rgt2* to regulate *RGT1*-mediated repression of nonpreferred carbon source genes. Like *HXK2*, *STD1* is also involved in glucose-induced repression of alternative metabolic pathways [48]. Interestingly, however, deletion of this gene typically leads to higher rates of growth [46],[49] and a disruption of gene regulation that is anticorrelated with that of a strain in which *HKX2* has been disrupted [47].

**Figure 5:**
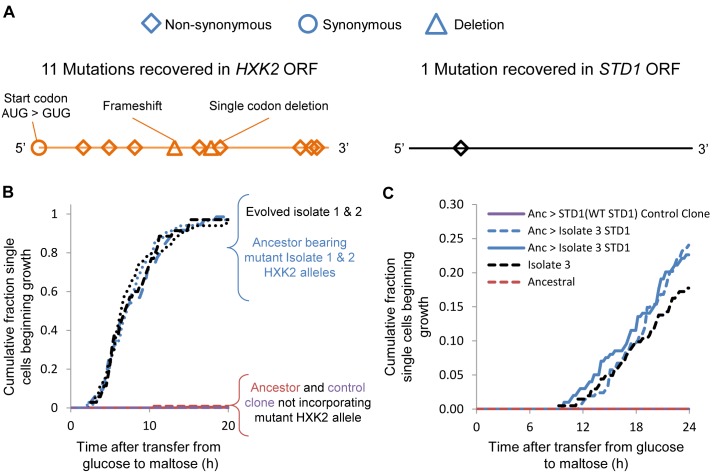
Mutations in global carbon catabolite repression genes give rise to diversified growth behaviors. Sequencing of the genomes of isolates with shorter lag phases isolated after repeated cycling between glucose and maltose medium revealed mutations in two genes, *STD1* and *HXK2* (see text for details). (A) Sanger sequencing confirmed the presence of multiple *HXK2* mutations and one *STD1* mutation in the evolved short-lagged strains. (B) Single-cell lag profiles of independent transformants of the ancestral strain bearing either WT (red and purple traces) or evolved (blue traces) *HXK2* alleles. Black traces correspond to the original evolved strains; the finely dotted line is Isolate 1 and the coarsely dotted line is Isolate 2. (C) Same as (B) but for the *STD1* allele identified in Isolate 3. Shown in blue are two independent transformants bearing Isolate 3's *STD1* allele.

Sequencing of *HXK2* and *STD1* in the 36 different evolved isolates revealed that *HXK2* was mutated at different positions in all but one of the different populations that showed shorter lag phases, whereas *STD1* was mutated only once (Figure 5A and Datasets S3 and S4). Isolates bearing *HXK2* mutations had significantly shorter single-cell lag phases in sudden glucose to maltose shifts than other isolates (12.72±2.85 versus 4.60±0.77 h for non-*HXK2mut* and *HXK2mut* strains, respectively) and were 26 times more likely to resume growth after a transition from glucose to maltose (Cox hazard coefficient = 3.26, *p*<10^−10^). Moreover isolates with *HXK2* mutations had significantly reduced population-level MaxR and increased GMR across the four carbon source environments (*p*<0.001). To confirm that these mutations were sufficient to confer comparable growth strategies to the evolved strains, we introduced the mutated *STD1* allele and two of the *HXK2* alleles into the ancestral S288c genome (Materials and Methods). The mutations phenocopied the behavior of the evolved isolates (Figure 5B,C). Furthermore, reverting the mutation back to WT in the evolved clones had the opposite effect, restoring wild-type growth patterns and fitness. Control strains bearing the same dominant marker but that did not incorporate the intended allele all behaved as the parental strain (Figure 5B,C and Datasets S3 and S4). Taken together, these findings reveal how simple mutations in carbon sensing pathways can tune the length of the lag phase in both gradually and suddenly changing environments.

### Evolved Mutants Display Differing Degrees of Catabolite Repression

**Figure 6:**
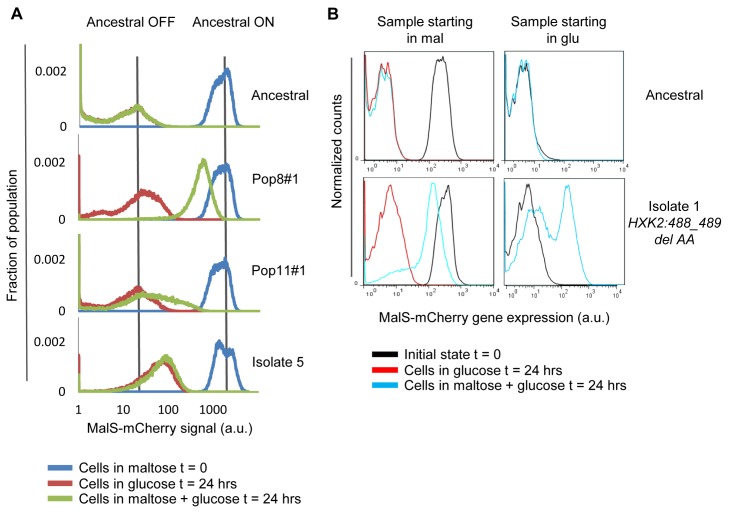
Mutants isolated after repeated cycling between glucose and maltose show altered catabolite repression of the *MAL* genes. (A) Flow cytometric analysis of four characteristic phenotypes that emerged after repeated glucose-to-maltose selection cycles show different degrees of glucose repression of the *MAL* genes. The different evolved strains with fluorescently tagged *MALS* genes were pregrown in maltose and then transferred to glucose media with or without maltose for 20 h of exponential growth, after which fluorescence intensities were measured using flow cytometry. (B) *MAL* expression in the evolved strains depends on the history of the cells. Initial state (black traces) of samples grown in maltose (left) or glucose (right) are shown, and in blue and red are the same cultures' expression levels after 20 h of growth in glucose alone or in a mixture of glucose and maltose. Note that the *MAL* expression level in the glucose/maltose mixture clearly depends on the history of the cells; the ancestral strain does not display this hysteresis.

To determine the molecular mechanisms giving rise to the altered growth characteristics in evolved isolates, we examined whether the mutants display altered *MAL* gene regulation. Flow cytometry measuring the fluorescence of the *MALT*-*YeCitrine* and *MALS-mCherry* reporter constructs revealed that many short-lagged mutants showed reduced catabolite repression of the *MAL* genes (indicated by “leaky” *MAL* gene expression in glucose), possibly explaining why they have shorter lag phases in sudden glucose-to-maltose transitions (Figure 6A). Furthermore, the degree of leaky background expression correlated with high fitness (GMR) in variable carbon environments and was inversely correlated with the MaxR and lag-phase length (Figure S5A and Dataset S2). Although the *MAL* genes are not necessary *per se* for growth in alternative carbon sources like ethanol or galactose, this correlation indicates that the leaky expression of the *MAL* genetic reporter relates more generally to a partial loss of glucose catabolite repression.

Most interestingly, in contrast to the leaky expression we observed in glucose media alone, we found that when maltose was added to glucose media, some *HXK2* mutants expressed their *MALT* and *MALS* genes to a great extent (Figure 6A and Figure S5). Deleting the *MAL* activator protein MalR (Figure S5D) did not affect leaky background expression but did relieve high levels of *MAL* gene expression in maltose and glucose-containing media, indicating that the *MAL* genes were being induced by the presence of maltose despite the glucose present in the media. There was significant variability in magnitude between isolates, and further between isogenic cells in the same population (Figure 6A and Dataset S2). However, within single cells, the average MalT and MalS signals were expressed to similar extents, implying that the entire *MAL* system was activated (Dataset S2). The different *HXK2* mutants show a wide range of *MAL* expression levels and expression noise, suggesting that the different mutations have distinct effects on catabolite repression.

### Different Degrees of Catabolite Repression Vary the Speed of Transcriptional Reprogramming and Allow Stochastic Sensing of the Environment

We reasoned that this heterogeneous *MAL* expression could serve as a model to elucidate how evolved isolates maintain steady fitness levels in more gradual diauxic shifts between glucose and maltose. To address this, we used both population- and single-cell analyses to track *MAL* gene activity (using fluorescent reporter constructs) and growth. Figure S5B and S5C and Movies S6, S7, S8 show the result of a time-lapse microscopy experiment of one isolate's cells growing in the presence of glucose and maltose. This experiment suggested that cells in the population switch stochastically between *MAL*-active and *MAL*-repressed states. Moreover, flow cytometry of a population of cells indicated that in the absence of glucose repression, similar to other positive feedback-driven networks in microbes [11],[30],[50], the *MAL* genes of these mutants show hysteresis (history dependence). That is, cultures that are inoculated into maltose/glucose media with repressed *MAL* genes display fewer induced cells than populations inoculated with *MAL* genes induced, even after ∼10 generations of growth (Figure 6B). We found the extent of this hysteresis was widely variable across the different *HXK2* mutants, with wide variability in the rates at which populations switch from induced to uninduced (ON to OFF) and vice versa (Figures 6B and S6 and S7 and Dataset S2). The per-generation switching rate from ON to OFF and OFF to ON for *HXK2* isolates depended on the initial carbon source (maltose or glucose) and the strain's genotype (two-way ANOVA *F* = 27, *p*<0.001; see Figure S7). The magnitude of expression of *MAL* protein for these strains exceeds the generation time, indicating that newborn cells inherit and propagate the *MAL* activity state from their respective mother cell (Figures 6B and S5, S6, S7 and Movies S6, S7, S8). These results imply that the different *HXK2* alleles led to varying rates of switching between *MAL* induced and repressed states.

The variability in *MAL* gene regulation has a profound effect on growth rate (Figure S8). For example, the magnitude of population-level *MAL* gene expression in maltose/glucose medium is anticorrelated with the population-level growth rate (Figure S8A), suggesting that the expression of *MAL* genes in medium containing glucose comes at a significant fitness cost. This cost is dependent upon an intact *MAL* activator, which drives *MAL* gene expression via positive feedback regulation (Figure S8B) [44]. Further single-cell analyses of one isolate confirm that genetically identical cells with transcriptionally active *MAL* genes grow at slower rates compared to cells that keep their *MAL* genes inactive (Figure S8C). Although induced cells suffer a fitness defect as long as glucose is present, they show a much reduced or even absent lag phase when glucose is no longer available, increasing their fitness during this transition phase (Figure S8D,E). In diauxic shift conditions, this bimodal gene expression state leads to a diversified growth strategy that distributes the costs and benefits of expressing the genes involved in alternative carbon source metabolism across individuals, allowing the population to maintain steady fitness levels in both stable and variable environments.

### A Mathematical Model Predicts Which Conditions Favor Specific Carbon-Catabolite Gene Regulatory Strategies

The isolates resulting from our evolution experiment and subsequent population- and single-cell-level analyses indicate that cultures can generally evolve in two directions. In one case, a “glucose-specialist” phenotype emerges, with faster adapted growth rates in glucose together with long lag phases upon a switch to a different carbon source. Alternatively, generalist phenotypes evolve with shorter lag phases in maltose, at an apparent cost to MaxRs in glucose. Furthermore, increased leaky expression of the *MAL* genes in short-lagged mutants is anticorrelated with MaxR in glucose, and positively correlated with reduced lag phases and fitness variability during diauxic shift (Figure S5A). This further suggests that a molecular cost is paid in order for cells to be prepared for sudden environmental changes (Figures 6 and S5, S6, S7, S8). We reasoned that the predominant growth strategy would be shaped by both (1) how often the culture must switch from stable glucose environments to alternative carbon sources and (2) the relative duration of these environments [1],[27],[34].

To test this, we first developed a stochastic model of the growth characteristics of population-level behavior based upon a culture of single cells escaping from the lag phase. In the model, each cell of a given strain's population is assigned a time *tau* corresponding to the point at which the cell will begin growth in maltose. The distribution of *tau* for a given strain in maltose is equivalent to the cumulative lag time distribution measurements (as reported in Figure 3A). After the time exceeds *tau*, the cell begins growth at the strain's specific growth rate in maltose. In glucose environments, we simulated that cells would grow without a lag phase at the measured MaxR. At any given time, the sum of the growing and nongrowing cells equals the total population growth as it escapes from the lag phase (Materials and Methods).

**Figure 7:**
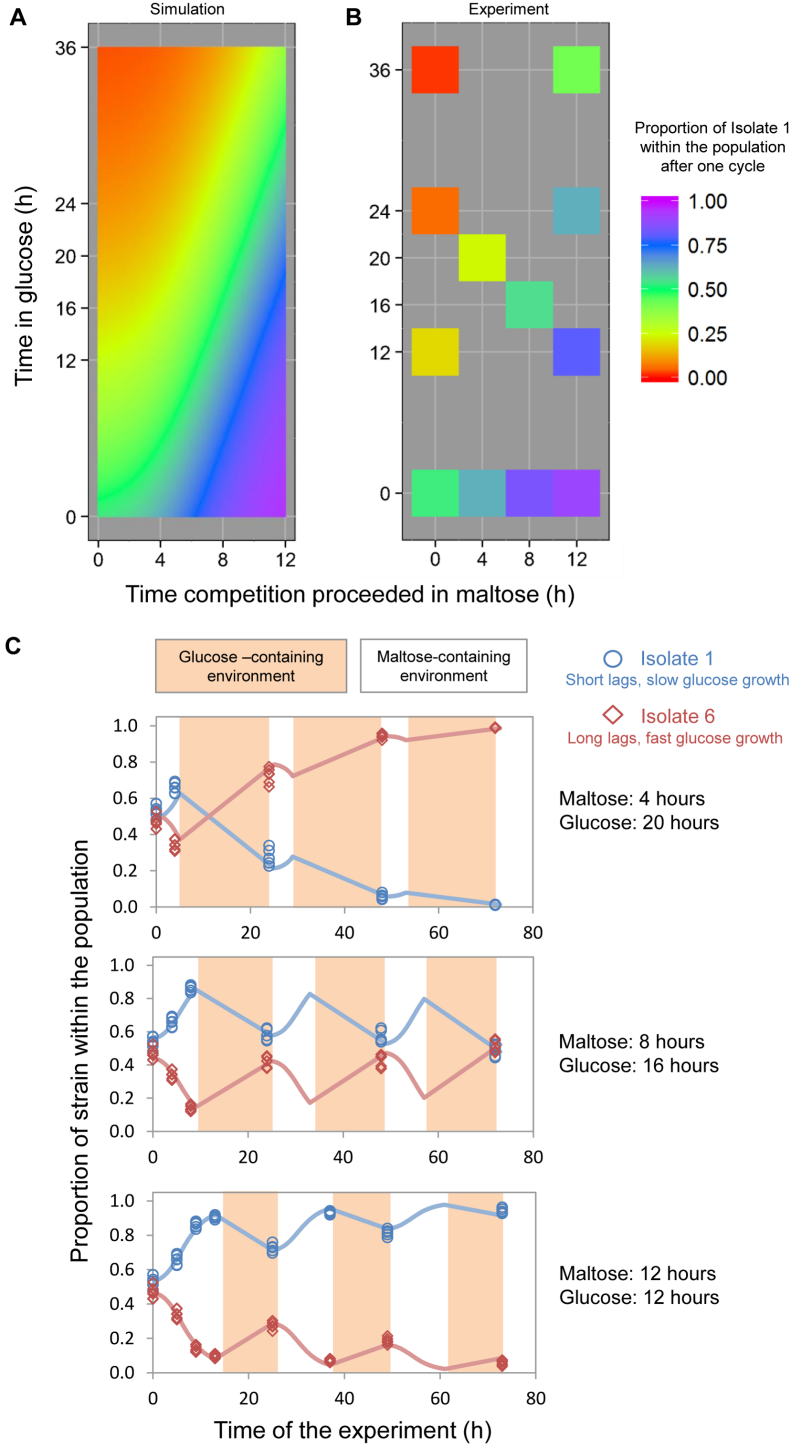
The tradeoffs associated with different levels of catabolite repression depend upon the frequency and duration of environmental change. Shown are modeled growth characteristics and experimental results of a head-to-head competition between Isolate 1, a strain with reduced catabolite repression, short lag phases, and slow rates of growth in glucose, and Isolate 6, a strain with stringent catabolite repression, long and heterogeneous lag phases but high growth rates in glucose, under varying regimes of glucose-to-maltose shifts. (A) Modeled fitness landscape predicting the relative abundance of short-lagged Isolate 1 relative to the long-lagged parental strain. The model uses experimentally determined lag phase distributions and adapted growth rates for each strain in maltose or glucose (see main text and Materials and Methods for details). (B) Heat map (same color scale as in A) of experiments where the two isolates were competed for different lengths of time in maltose and glucose (12 different regimes, with each 5–6 repetitions). The strains were grown for 20 h in glucose before being transferred to maltose for 1 h, and then mixed together to begin the competition experiment. (C) The cultures represented by the points in panel (B) corresponding to 4 h∶20 h, 8 h∶16 h, and 12 h∶12 h maltose-to-glucose cycles were repeatedly subjected to the same regime of maltose-to-glucose changes to track relative abundance of the competing clones over time. Dots represent experimental observations, and lines represent modeled growth characteristics of these strains.

The model allows prediction of the clonal interference patterns that would result between competing strains with different lag characteristics across various maltose-to-glucose switching regimes. For example, the heat map in Figure 7A illustrates how Isolate 1, a “carbon source generalist” *HXK2* mutant with short lag phases, would compare in direct head-to-head competition with Isolate 6, a “glucose-specialist” strain with ∼28% faster growth rate in glucose compared to Isolate 1 but with considerably longer and more heterogeneous lag phases (as shown in Dataset S2). The model predicts that an environment that consists of a single shift from glucose to maltose will result in Isolate 1 growing rapidly to high relative frequencies in the population due to its very short lag phase. However, during prolonged growth in glucose, Isolate 1 grows more slowly and thus is rapidly outcompeted. Importantly, certain regimes of maltose-to-glucose shifts are predicted to result in a stable abundance of each isolate relative to the other (green region in Figure 7A).

### Experimental Validation of the Model Reveals How the Frequency and Duration of Glucose and Maltose Environments Shapes the Evolution of the Lag Phase and Growth Rates

We tested the model in a set of experiments where two strains with different lag characteristics were competed in different carbon switching regimes. In a first set of experiments (Figure 7B), we measured Isolate 1 and Isolate 6's abundance relative to one another in single shifts from glucose to maltose, with varying duration of growth in glucose and in maltose. The cultures were grown initially in glucose so that they would have a lag phase upon switching to maltose, or alternatively carry on at steady-state growth rates in glucose. The results confirm the trend suggested by the model: under regimes with longer periods of maltose growth, Isolate 1 shows the highest competitive fitness; conversely, with increasing time in glucose, the fast-growing Isolate 6 performs better. Second, to examine if the model could predict strain performance over multiple cycles of glucose-to-maltose shifts, we carried the experiment forward for another two cycles for the cultures growing under the 4 h∶20 h, 8 h∶16 h, and 12 h∶12 h maltose-to-glucose switching regimes (Figure 7C). The results confirm that different regimes of environmental change result in drastic changes in the frequency of the two competing genotypes within the total population. For example, in the top panel of Figure 7C, when the period of time in glucose exceeds the time in maltose, Isolate 6 comes to dominate because the benefits of faster growth rates in glucose outweigh those of shorter lag phases in maltose. By contrast, as the length of time in maltose increases, the short-lagged Isolate 1 outperforms the slower switching strain despite lower rates of growth in glucose (Figure 7C, bottom panel). Note that the competitor populations depicted in the middle panel remain at relatively equal frequencies when the culture undergoes cycles of 8 h of growth in maltose followed by 16 h of growth in glucose.

Interestingly, in the 12 h∶12 h regime, we observed that Isolate 1 grew ∼10% more slowly over the glucose leg of the cycle compared to our measurements of the same strain in steady-state conditions (Dataset S8)—thus growing ∼40% more slowly than the Isolate 6 competitor during glucose growth. Across the entire 24-h period of the glucose to maltose cycles, we measured that this slower rate of growth in glucose reduces the average growth rate of this strain by about 5% (Dataset S8). The reduced rate of growth in glucose indicates that Isolate 1 paid a considerable cost upon reintroduction to the glucose-containing environment. This result further supports conclusions from the experiment reported in Figures 2 and S8 where commitment to maltose resulted in slower growth rates for individual cells.

Taken together, these results demonstrate that the duration and frequency of carbon environments shape the fitness of two of the archetypal phenotypes recovered from the evolution experiments. Environmental regimes with long periods of growth in glucose relative to the duration of time in maltose will favor the growth of the specialist phenotype, with stringent catabolite repression and high growth rates in glucose, but long lag phases upon switching to a different carbon source. By contrast, more frequent shifts of carbon source and longer periods of growth in maltose will favor the growth of strains with less stringent catabolite repression, resulting in short, homogeneous lag phases and slow growth in glucose.

## Discussion

### “Why the Lag Phase?” An Old Problem Revisited

When Monod (1941) [26] first described the diauxic shift and the corresponding lag phase, he was frustrated by why a culture would reach such slow growth rates despite the abundance of alternative carbon sources. Monod realized that the lag phase was due to the time required for transcriptional reprogramming, which in turn propelled research into the molecular mechanisms underlying gene regulation. However, surprisingly little attention was given to how this transcriptional reprogramming affects growth rate. Our experiments show that the speed with which metabolic genes are reprogrammed in the face of changes in carbon availability is a highly variable and evolvable trait. Strains can maintain high fitness in stable glucose conditions by tightly repressing the costly expression of genes needed for growth in alternative carbon sources. However, this results in slow transcriptional reprogramming when glucose is depleted, leading to reduced fitness during the adaptation to a less-preferred carbon source. By contrast, less stringent catabolite repression and “stochastic sensing” strategies come at a fitness cost in stable conditions, but allow quick and more uniform adaptation, which in turn can lead to higher fitness during the transition phase.

### Similarities and Differences Between Natural and Laboratory Evolution

It is tempting to speculate that feral yeasts are faced both with relatively stable as well as variable carbon supplies, and are therefore subjected to pressure that is similar to the selection in our directed evolution experiments. Moreover, the mutants that we isolated after experimental evolution closely resembles the diversity found in natural isolates, both regarding differences between strains as well as differences between single cells within populations (Figures 1H, S3, and S4D). Furthermore, we observe that mutants isolated after selection in variable environments appear to be constrained by opposing selection for maximal reproductive fitness in stable glucose environments versus adaptability in variable environments. These results are consistent with theories of phenotypic tradeoffs associated with optimality at specific tasks versus the capacity to adapt to new environments [4],[6],[9],[17]–[20]. It is notable, however, that we do not observe the same level of direct tradeoffs between MaxR and lag phase across the strains reported in Figure 1 as we do in the evolved isolates. Given the allelic diversity of these strains, such a result is not unexpected. For example, previous evolution experiments have observed that common phenotypic tradeoffs, such as that between growth rate and carrying capacity, become undetectable with increasing genotypic distance [7]. We therefore speculate that single mutations that give rise to tradeoffs in the evolved isolates could potentially be compensated by additional mutations that might increase overall fitness or that other evolutionary paths are followed in more complex natural environments [51].

### The Lag Phase from an Ecological Viewpoint

The natural strains and evolved mutants with short lag phases show similar differences in lag duration and fitness variability. More specifically, strains with short lag phases show similar fitness levels across different environments, a phenotype referred to in the ecological literature as a “generalist” strategy to indicate that these organisms thrive equally well in different environments. It is also worth noting that the *HXK2* and *STD1* mutants showed increased fitness both in gradually changing conditions (LG, LG + maltose, and LG + galactose) where the preferred carbon source is gradually depleted, as well as in conditions where the cells experience sudden changes in carbon availability (Figure 4 and Dataset S2). In ecological theory, both of these “fine-grained” and “temporally fluctuating” environments are predicted to favor generalist growth strategies, with reduced variability in fitness across different environments [1],[4]–[6],[27],[39],[40]. However, in contrast to our results, most experimental evolution studies tend only to find niche specialization (i.e., higher rates of growth on one carbon source or the other) [52],[53]. It seems likely that these studies uncovered specialist phenotypes because the selective pressure to improve adapted growth speeds on particular carbon sources was greater than the pressure to reduce lag phases or increase switching rates between carbon sources [28],[54].

The mathematical model and experiments reported in Figure 7 provide further insight how these strategies are shaped and favored by the environment. These analyses illustrate how different regimes of environmental change favor the growth of either specialist strains with stringent catabolite repression or alternatively generalist strains with leaky or even stochastic expression of genes involved in metabolism of different carbohydrates. More broadly, these results confirm theoretical analyses and experimental reports that while stable preferred environments will promote slow switching rates, frequently changing environments can favor the growth of phenotypes with high adaptability, even at the cost of slower rates of growth in preferred environments [1],[8],[27].

### Single-Cell Heterogeneity as a Bet-Hedging Gene Regulation Strategy

Interestingly, apart from revealing differences in growth strategies between genetically different yeast strains, our results also illustrate how genetically identical cells within a population can also show different growth behaviors that can help to optimize a strain's fitness in variable conditions. Specifically, the results show that an organism can maintain competitiveness by allowing only a fraction of the population to adapt to a new environment. This subpopulation of cells contributes some progeny to exponential growth while keeping others dormant (Figures 1G, 3A, and S3). Although they contribute no progeny in the new environment, these uninduced cells appear to have an advantage upon reintroduction of the previous environment (Figure 2), increasing overall mean growth rates (Figure 7). Such growth strategies have commonly been investigated for the model of seed germination in annual plants, a system that in many ways bears semblance to our model system of the lag phase [42],[55],[56]. Heterogeneity within isogenic populations bears further similarities to other microbial systems that lead to variable physiological states between isogenic individuals in the same population. For example, heat-shock resistance [19], the variable timing of meiosis [57], or the switch-like commitment to mating [58] in yeasts likely underlie a cost-benefit strategy that increases fitness in the long term. Indeed, these systems are costly to implement [19],[57],[59] but offer fitness advantages in stressful environments. However, despite receiving much attention in the literature, few studies have systematically quantified how heterogeneous individual-level behavior can affect evolutionary outcomes [27],[33],[55],[56].

### Mutations in Global Regulators Control Growth Strategies

Our evolution experiments demonstrated the flexibility of growth strategies and also revealed that simple genetic switches in catabolite repression can regulate lag duration (Figures 4–6). Specifically, *HXK2* mutations appear to increase fitness in lag phases in glucose-to-maltose shifts in part by allowing leaky expression of *MAL* genes (Figure S5). The repression of other genes involved in alternative carbohydrate metabolism is also likely relieved because the fitness of the mutants also improved in mixed carbon conditions different from those used in the selection scheme. This conclusion is supported by whole-genome gene expression profiling of a targeted *hxk2* deletion [47], where genes involved with respiration and alternative carbon source utilization are significantly up-regulated. Although null mutations in *HXK2* are known to relieve catabolite repression in S288c, the potential for more subtle mutations in this key regulator to allow “tuning” of switching rates between alternative carbon sources has not been explored extensively [45],[52],[53],[60],[61]. Furthermore, in the case of the *STD1* allele, we observe reduced growth rates, which is the opposite effect to that usually observed in high-throughput studies, where null *STD1* alleles grow with higher fitness than the WT S288c strain [46],[49]. Taken together, this aspect of our results suggests that regulators such as *HXK2* sit at an apical position in the regulation of cellular physiology, allowing adaptive reprogramming of cellular fitness strategies in times of environmental change.

### “Stochastic Sensing” Is a Gene Regulatory Strategy That Falls Between Bet-Hedging and Environmental Sensing

Interestingly, some of the short-lagged isolates show a high degree of heterogeneity in *MAL* expression within a population (Figures 6 and S5). Specifically, in medium containing both glucose and maltose, some *HXK2* mutants exhibit a striking multimodal state, where *MAL* genes in individual cells are expressed to varying extents ranging from repressed to induced. This behavior emerges because the rate of switching between ON and OFF is slower than the generation time, allowing newly budded cells to inherit their *MAL* expression state from the mother cell (Figures S6 and S7). This epigenetic behavior is due to the structure of the *MAL* genetic circuit, which induces via positive feedback (Figures S5D and S8B) [11],[30],[44],[50]. Furthermore, it appears that distinct *HXK2* mutations can set different “energy barriers” for transitions between induced and uninduced states (Figures 6A,B, S6, and S7). Although such “stochastic switching” networks have been reverse engineered (for example, [27]), and shortened lag phases observed in natural selection [28],[54], no studies have found that mutations in global regulators can give rise to such a wide array of diversified gene regulation strategies.

The simple genetic architecture of the *MAL* system has allowed us to closely examine the costs and benefits of different levels of catabolite repression and the outcomes of stochastic gene regulation [3],[10],[11],[20],[62],[63]. The stochastic nature of the transition between *MAL* activation and repression results in diversified growth behavior that appears to be a bet-hedging strategy. However, maltose must be present to induce the positive feedback necessary for the high levels of *MAL* gene expression shown in Figures 6 and S5, S6, S7, and thus this environmental dependence does not satisfy the most stringent criteria for bet-hedging [33]. Even in the case of stable, constant glucose environments, the low leaky expression of costly nutrient assimilation genes could be viewed as a mechanism of “stochastic sensing,” a term first used by Perkins and Swain (2009) [37] to describe predictive microbial networks [35],[36]. More recently, Arnoldini et al. (2012) [34] demonstrated analytically that combinations of sensing and stochastic switching strategies are evolutionarily stable when environments provide partially reliable signals about future events. Given that such positive feedback-driven circuits are widespread in microbes, it is likely that nutrient assimilation pathways act as basic sensing tools to maintain long-term fitness in changing environments, without the need for complex sensing and signaling systems [1],[21],[27],[29],[30],[34],[35],[37],[64],[65].

### Conclusions

Taken together, our results show that individual-level heterogeneity in gene regulation and growth has strong genetic determinants. The speed of metabolic reprogramming in the face of environmental change is a highly regulated trait, and populations can implement catabolite regulatory strategies that fall between traditional sensing/signaling cascades and stochastic switching mechanisms. Specifically, stringent catabolite repression seems favorable in relatively stable environments, whereas less stringent regulation, or even stochastic sensing strategies can increase fitness in variable conditions where cells often need to switch their metabolism. We speculate that similar principles and emergent (epi)genetic switches likely also contribute to other gene regulation systems, including in human diseases involving clonal growth, such as microbial pathogenesis and cancer.

## Materials and Methods

### Strains and Media Used

Standard protocols were used for routine *S. cerevisiae* strain propagation [66]. A specially engineered maltose-prototrophic S288c strain, bearing a functional *MAL* regulator allele (*MAL63*) in place of *MAL13* on chromosome VII [67], was engineered to have a low petite frequency by rescuing a frameshift mutation in *SAL1* to reduce the high petite frequency that occurs after extended growth on glucose (Text S1 and [68]). Other feral strains were part of the SGRP collection [69]. Special attention was given to standardization of pregrowth conditions, in particular to avoid cells that would experience carbon depletion prior to transfer to maltose for lag phase measurements. Specifically, this entails keeping cultures at low densities throughout the experiments. Moreover, where appropriate, care was taken to measure steady-state conditions (where the growth speed of the population was stable). Please refer to specific experimental details provided in Text S1 for the precise conditions for each experiment.

### Population-Level Growth Rate Measurements Using Bioscreen C

Cells from a turbid culture grown in YPD for 14 h were inoculated to a final density of 1×10^5^ (haploid S288c) cells per ml in 150 microliters of YP media containing the appropriate carbon source and allowed to grow in the Bioscreen C (Growthcurves USA) at 30°C and continuous medium-amplitude shaking until stationary phase. All media for growth rate measurements were prepared starting from the same batch of double concentrated YP medium (20 g/l yeast extract, 40 g/l bacterial peptone), which was supplemented with an equal volume of filter-sterilized sugar solutions to generate 1× YP medium containing the required mixture of carbon sources to obtain HG (30 g/l glucose), LG (5 g/l glucose), LG + Gal (5 g/l glucose and 25 g/l galactose), or LG + Mal (5 g/l glucose and 50 g/l maltose). We found that variation in osmolarity is a significant factor affecting the lag phase, and we therefore supplemented LG medium with 0.14 molar sorbitol to match the osmolarity of the HG, LG + Mal, and LG + Gal media. All media were divided into smaller batches that were kept frozen until the day of use.

All growth measurements represent the averages of at least three biological replicates. In general, growth measurements were highly reproducible, with standard errors generally below 5% of the measured growth rates. Standard errors are reported in detail in Datasets S1, S2, S3, S4, S5, S6, S7, S8. Growth of populations of *S. cerevisiae* were measured by OD600 readings every 15 min in the Bioscreen C automated OD meter at 30°C with constant medium amplitude shaking. This plate reader uses 100-well microcultivation of microbial cultures covered by a heated lid to prevent evaporation. R and MS Excel software were used for all data analyses. Briefly, all growth curves were smoothed using R's smooth.spline function, and then the first derivative of the log-transformed smoothed data was plotted as a function of population size between 0.15 and 0.75 OD600 units, corresponding to 1.0×10^7^ to 5×10^7^ haploid S288c cells per ml (see Datasets S6 and S7). These values are linearly correlated with cell density, and further correspond to lowest and highest OD at which we were able to obtain reproducible measurements, and further capture most of the active growth phase of the cultures before other noncarbon growth resources become depleted.

Reported maximum growth rates were calculated as the slope of a linear regression model to ln-transformed OD600 values between 0.15 and 0.3 A.U. To calculate GMR, we used R's splinefun function to determine the amount of time *t* that the culture spent between interpolated OD600 values of 0.15 and 0.75; the GMR is thus equal to ln(0.75/0.15)/*t*. This interpolation approach significantly reduces the coefficient of variation between biological replicates (error) compared to a simpler approach that would only use the raw OD measurements. These statistics were further analyzed to determine relative variability across the different environments (see Datasets S1 and S2).

Note that the GMR is an average growth rate that could be calculated for many different initial and final population densities. Here, we use the GMR to reflect the average growth rate across the linear range of our spectrophotometer (i.e., between O.D. 0.15 and 0.75). This is a relatively wide interval that comprises the initially high growth rate in glucose, the lag phase, and the resumption of growth on the new carbon source (Figure 1A,B; Materials and Methods). This interval is easy to standardize and yielded a high reproducibility (Datasets S1 and S2). However, to explore the role that the lag phase played in the GMR, we also calculated average growth rates over other more narrow windows centered on the middle of the lag phase (defined as the point where the culture reaches a minimal growth rate). These measures correlate strongly with the GMR. For example, the narrowest possible window—the minimal growth rate (reported in Datasets S1)—explained 55% of the variance in GMR for the SGRP strains that had lag phases (Dataset S1, R^2^ = 0.55, p<0.001). Computing the average growth rate across a wider custom-drawn window encompassing 0.1 to 1 doublings prior to and 0.1 doublings after the minimal growth rate explained from 66% to 79% of the variance in GMR (*p*<0.001). In summary, the GMR in between OD 0.15 and 0.75 seems to be largely influenced by the actual lag phase. Moreover, calculating the GMR across more narrow intervals around the center of the lag phase is much more complex, is not always possible for all growth conditions where strains do not always show a local minimal growth rate, and yields similar results. Further details and example raw data and analyses are available in Text S1.

### Single-Cell Measurements of Lag Phases, Growth Rates, and Gene Expression

We devised a system that traps cells between a coverslip and an agar pad containing media necessary for growth. This allowed continuous monitoring of cellular growth for long periods of time using an inverted automated Nikon TiE fluorescence microscope placed in a temperature-controlled incubator. A 60×, 1.40 NA oil immersion lens was used to monitor up to 120 XY positions per experiment, and lag measurements were made using the microscope's automated Z-plane focusing.

Lag phases for a given strain become longer as the culture grows for longer periods of time in glucose (Figure S2A). Thus, for analysis of lag phases, we varied the length of time that cells were grown in glucose to regulate the severity of single-cell lag phases. Apart from the results reported in Figure S2A, we either grew populations in glucose for 6 h (reported in Figure 1) or 20 h (reported in Figure 3—the length of time cells grew in glucose during the evolution experiment). After growth in this glucose environment, we transferred cells by two brief (2 min at 1,250×g) centrifugations and resuspension in maltose-containing media. Cells were then transferred to the custom growth chamber (see Text S1) and transferred to the microscope for analysis. Under the microscope, brightfield imaging proceeded every 15 min. After the experiment, cell budding events were scored manually as the hour at which the first morphological change leading to a new bud occurred, or when an already existing bud began to grow. Examiners were blind to experimental conditions at the time of scoring, and separate investigators independently replicated results of preliminary analyses.

For statistical analysis of single-cell lag data, we used survival analysis: log-rank tests for pairwise comparisons between different WT strains or between ancestral strains and mutants, and a Cox proportional hazards test for datasets for which we had covariate information (see Text S1). For these latter analyses, single covariates were used (1 degree of freedom), and the most significant predictors were then used in paired analyses using other covariates (2 degrees of freedom). The majority of single-cell lag variance was explained by single covariates that reflected relative fitness (GMR) or fitness variability (Dataset S1).

For doubling time measurements presented in Figure 2, an initial fluorescence image was acquired, followed by brightfield imaging every 5 min. Mother cell doubling times were recorded as the time *t* taken for a cell to complete two cell divisions (equal to *t*/2). Brightfield images reported in Figures S5 and S8 were acquired every 5 min, with fluorescence images in the mCherry field every hour. Growth rate measurements of microcolonies reported in Figure S8C account for the fold change in area of microcolonies between 3 and 6 h after recording began (equal to Δ(ln(area))/3 h) [19].

### Population-Level Gene Expression Measurements

To measure the relative *MAL* expression reported in Figure 6 and Dataset S2, we pregrew cultures in maltose media (Figure 6A) or either maltose or glucose media (Figure 6B) and diluted them as exponentially growing cultures into a mixture of 2% glucose + 5% maltose in YP media to a final population density of 1–2,000 cells/ml and allowed them to grow for 20 h for final cell densities between 1–5×10^6^ cells per ml. Cultures were then centrifuged to concentrate cells, and these were frozen at −80°C in 1× phosphate buffered saline (Sigma-Aldrich no P5493) in 25% glycerol until flow cytometric analysis. The low densities at which these cultures grew did not measurably affect glucose concentrations (unpublished data). For gene expression measurements, cell samples were thawed on ice until analysis on a BD Bioscience Influx flow cytometer. mCherry signal detection used a 561 nm laser coupled to a 610/20 nm detector and YeCitrine signal detection used a 488 nm laser coupled to a 580/30 nm detector. R's flowCore package was used to first filter out ∼70% of events using a filter (curv2Filt) that selected the highest-density regions in side- and forward-scatter dimensions. mCherry and YeCitrine intensities for each filtered sample were stored as binned fluorescent measurements and summary statistics.

### Experimental Evolution and Selection Protocol

For each evolution experiment, the methods established by Lenski were largely followed [70]. All growth for the experimental evolution experiment was in 5 ml of YP media containing either 10% glucose or 20% maltose at 30°C on a rotating wheel. The protocol was followed for two founding S288c strains derived from the modified S288c strain (see above): AN296 constitutively expressed a YeCitrine marker [71], and AN148 contained fusion constructs of *MAL11-YeCitrine* (encoding a MalT) and *MAL12-mCherry* (encoding a MalS). Populations of ∼30,000 initial cells were grown exponentially for 20 h in 5 ml glucose YP to reach population sizes of ∼1–5×10^7^, and then cultures were centrifuged and resuspended in 20% maltose YP and then put back on the wheel for another 3 d until the populations reached high densities (∼5×10^8^ cells/ml, with a final population size of ∼2.5×10^9^). After each round of selection in maltose, we froze an aliquot at −80 for future analysis and resurrection. At the end of the selection experiments, we resurrected individual clones for analysis by diluting the populations to single colonies, and then restreaking random single colonies again to single colonies before phenotypic characterization and long-term storage at −80°C in glycerol.

### Malthusian Fitness Measurements

For the experiment reported in Figure 3B, we pregrew constitutively YeCitrine-labeled query strains (isolates 1, 3, and 4) in sextuplicate and mixed these 1∶1 with an mCherry fluorescently labeled S288c reference strain (AN74; see strain list in Dataset S4) exactly as in the selection protocol in 20% maltose YP media for 24 h. OD600s were determined and query cultures were mixed at a 1∶1 ratio between reference and query strains. Samples were frozen for initial ratio measurements, and initial population densities determined by diluting cells down such that 100–200 single colonies would grow in 2 d time on solid YPD plates. Thereafter, the exact protocol from the selection procedure was then followed: a 20-h growth in glucose, followed by 2 d of growth to high turbidity in maltose media. At the end, samples were frozen in 1× PBS + 25% glycerol for later analysis, and final population densities determined by plating as before. For flow cytometry analysis, see below.

We calculated the Malthusian growth rate w of the query as:
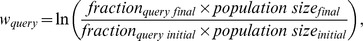
where N is the total population size determined by plating. Likewise w(reference) was calculated for the fluorescently labeled reference. The w(query)/w(reference) was taken as the fitness of the query strain. This value divided by the ancestral strain's fitness (calculated identically against the same reference) gives the fitness of the query strain. All fitness or relative growth rate measurements are the ratio of the query strain's fitness relative to the reference strain divided by the control query strain's fitness relative to the reference strain.

### Mathematical Model of Population Growth in Maltose-to-Glucose Cycling Environments

For a given culture of cells, the population total *N_total_* is equal to the sum of each *i^th^* growing and nongrowing cell. Within the population, there are J phenotypes (or strains) and each cell belongs to the *j^th^* strain or phenotype such that *N_totalj_* is the number of cells of the given phenotype or strain. At any given time, the proportion of cells in the *j^th^* phenotype is:
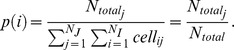



Each strain *j* in environment *k* has a specific growth rate equal to μ_j,k_.

The model assumes specifically that cells entering into glucose from maltose do not have lag phases. Cells that have no lag phase in environment *k* grow at steady-state growth rates; thus, at time *t* the total population size of strain *j* is determined by: 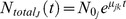 with total population size 
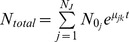
.

The model assumes specifically that cells going from glucose to maltose do have a lag phase. In a lag phase within environment *k*, cells of strain *j* have lag phase durations *tau* equal to a vector of lag phase durations drawn from experimentally determined distributions *dist(τ_j,k_)*.

In the lag phase, cells of strain *j* only begin growth at steady-state growth rate *μ(j,k)* when *t = τ_j,k_(cell_ij_)*.

Each cell_i,j_ has an initial population size equal to 1. Thus, in the lag phase the population size *N* for the *j* ^th^ strain at time *t* is the sum of growth of all cells *i*.
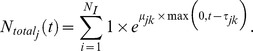



And the total populations size *N* at time *t* is the sum of all j competitors.
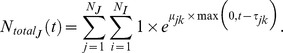



The model was implemented as a stochastic simulation in R (functions and scripts available upon request). Time was iterated in glucose and maltose environments in 0.1-h increments. A total of 50,000 cells were chosen as initial population sizes for maltose lag phase simulations, and each cell was given a lag time tau and a status (1 or 0) that indicated whether this cell would begin growth within 24 h (the period of time for which we had data about lag time distributions for these strains). A normal distribution of *tau* was chosen for Isolate 1, and a uniform distribution for Isolate 6. After computing lag phase growth as a function of time in maltose, growth in the glucose environment was computed for each time point in maltose. The resulting matrices of population sizes for the two competitors were used to compute the proportion of each strain in the total population.

In the text and in Figure 7, *p(i)* is the only parameter reported. In Figure 7A, the plot reflects the initial conditions of the experiment pictured in Figure 7B and 7C, where the average initial proportion of Isolate 1 relative to Isolate 6 for the six independently competing populations was 0.53317 and the cells had been in maltose for 1 h prior to time point 0. See Dataset S8 for the exact parameters used in the model.

### Competition in Variable Maltose and Glucose Environments

To begin the experiment where strains were competed in various carbon switching regimes, six independent replicate populations of Isolate 1, a strain constitutively expressing YeCitrine, and Isolate 6, a MALT-YeCitrine and MALS-mCherry strain, were inoculated from turbid YPD overnight cultures into glucose media and allowed to grow for 20 h until they had reached population densities of <5×10^6^ cells per ml, such that both cultures would have lag phases upon transfer to maltose. The cultures were washed into maltose media, OD600 values measured, and then mixed 1∶1 and either kept in maltose or were transferred to glucose to generate the results shown in Figure 7B. This initial time point also served as the beginning of the glucose-to-maltose cycling populations shown in Figure 7C. Growth was at maximum 5×10^6^ cells per ml, and minimal population sizes for long periods of glucose growth were ∼20,000 cells. At each time point, cells were centrifuged and frozen at −80°C in 1× PBS + 25% glycerol for later analysis at the flow cytometer. We used the same glucose and maltose YP media and volumes in this experiment as in the selection procedure.

For analysis of competitions, 50,000 single-cell events were acquired by a BD Biosciences Influx flow cytometer. We used a 561 nm laser coupled to a 610/20 nm detector for mCherry, and for YeCitrine signal detection, we used a 488 nm laser coupled to a 580/30 nm detector. We used R's Flowcore package to identify subpopulations of unlabeled, YeCitrine-labeled, or mCherry-labeled cells using polygonal gates. We used control cultures of each competitor growing on its own in identical conditions to those of the competition to determine the fraction of events that were incorrectly determined to be one competitor when in fact they were from the other. In general these error rates were below 1/5,000 events.

### Whole Genome Sequencing and Variant Calling

Selected samples were whole-genome sequenced using Illumina HiSeq 2000 with 500 bp inserted library. Quality assessment of resulted short reads was performed using FASTX-Toolkit (http://hannonlab.cshl.edu/fastx_toolkit/index.html). After removing the low-quality reads (below Q30) and adaptors, pair-end reads were then mapped onto the reference *S. cerevisiae* genome (S288C, version genebank64) using Burrows–Wheeler Alignment [72]. Default settings were used except the maximum edit distance was set to 0.01 (−n 0.01). The MarkDuplicates command in Picard (http://picard.sourceforge.net/) was used to remove the reads that mapped to the same positions in the reference genome (PCR duplications). Consensus single-nucleotide variations (SNPs) and small insertions and deletions (Indels) were called for each chromosome using SAMtools and GATK [73],[74]. Default settings were used, except the maximum read depth in SAMtools was set to 150× (−D 150). The generated SNPs and Indels were then filtered to minimize the false positive mutation calls. First, SNPs and Indels lying in low complexity sequences (such as telomeric, subtelomeric, transposon, repeat regions, etc.) were filtered out. Second, mutations with a total read depth below 20× were discarded. Third, SNPs and Indels with a quality score below 30 were removed. Fourth, mutation calls were only kept when at least 80% of the reads were positive for the SNP sites. Only the SNPs/Indels that were verified by both GATK and SAMtools were kept as confident sites. The lists of SNPs/Indels were then annotated by in-house Perl scripts with the yeastgenome database [75]. CNV-seq [76] was used to identify consecutive regions along the chromosome that show abnormal log2-ratios, which indicated the potential copy number variation (CNV). Only regions larger that 1 Kb were considered as CNV regions.

### Genetic Complementation Analysis

Mutations identified by whole genome sequencing that lead to nonsynonymous or frame-shifted protein products in *HXK2* and *STD1* were first confirmed with dye-terminator Sanger sequencing. To test whether the mutations caused the observed phenotypes, we first integrated a dominant (*KANMX*) marker downstream of the ancestral and evolved alleles. This marker, including the upstream coding region containing the mutated or WT allele, was used as a template for PCR, which was then transformed via homologous recombination into the corresponding loci in the evolved or ancestral clones. The mutations were subsequently confirmed using Sanger sequencing, and the phenotypes of the genetically transformed strains was compared to that of strains bearing the same KANMX marker at the same locus, but lacking the mutation.

## Supporting Information

Dataset S1 **Overview of the growth rate and lag measurements for 16 feral yeast strains.** The three subtables (A–C) in this MS Excel document summarize the data we used to determine lag phase characteristics in the SGRP yeast strains. (A) This table includes population-level growth rate measurements of the wild strains, including MaxR, GMR, MaxR-Normalized GMR, the minimum growth rate detected in each experiment, and the point in the growth curve at which the locally minimal growth rate was observed. Samples where the minimal growth rate is approximately equal to 0.75 indicates that there was no lag phase, as this was the maximal OD600 value over which growth rates were analyzed (see Experimental Procedures). Variates of across-environmental variability used as covariates for Cox proportional hazards analysis in R. (B) This table includes all censored single-cell measurements made for the 16 yeast strains reported in the text, used to generate, for example, Figure 1G and 1H. The single-cell lag phases of some strains were measured following identical procedures using galactose or ethanol in place of maltose. (C) Each strain's censored single-cell lag data going from glucose-to-maltose were paired with covariate data and used as input for R's Cox proportional hazards analysis. The table summarizes the output of this analysis for each of the covariates examined. Each covariate was compared alone to single-cell lag time observations (1 degree of freedom), and then we used a couple covariates in combination with the other covariates to see how much more variance could be explained by the combination of factors. The fold likelihood of lag phase escape of samples' maximal or minimal covariate values is calculated based upon the equation exp(beta×min)/exp(beta×max). Hazard coefficients of negative value are factors that tend to correlate with average single-cell lag length (final column, outside the black line). In general, measures of growth rate (GMRs) variability explain most of the variance in single-cell lag time distributions.
(XLSX)

Dataset S2 **Overview of the growth rate and fluorescence measurements for the evolved strains.** This dataset relates to data presented in Figures 3, 4, and 6. (A) This table includes population-level growth rate measurements of evolved isolates, including MaxR, GMR, MaxR-Normalized GMR, the minimum growth rate detected in each experiment, and the point in the growth curve at which the locally minimal growth rate was observed. Samples where the minimal growth rate is approximately equal to 0.75 indicates that there was no lag phase, as this was the maximal OD600 value over which growth rates were analyzed (see Experimental Procedures). (B) All censored single-cell lag data of cells going from glucose to maltose for the populations isolated from the evolution experiment; see Dataset S1B. (C) This table includes the summary of a log-rank survival analysis in R using the “survdiff” function (see Scripts.R and Experimental Procedures for more details). The single-cell lag phases of isolates from the experimental evolution experiment were compared to that of the ancestral strain. For the Isolates 1–6, samples were grown for 22 h in glucose before transfer to maltose. Because this long period of growth results in longer and more heterogeneous lag phases, comparison with the ancestral strain was more difficult for the other *MAL* gene fluorescently labeled cultures. Thus for the rest of the *MAL* gene fluorescently labeled populations, we compared survival for samples after only 6 h of growth in glucose. All comparisons were made against the ancestor growing in identical conditions. Most strains have survival that is higher than expected compared to the ancestor, except a few that have even longer lag phases. (D) *MAL* gene fluorescence measurement summaries computed using R's Flowcore package.
(XLSX)

Dataset S3 **Overview of mutations in experimental evolution populations.** This dataset relates to the data presented in Figure 5. Samples were dye-terminator Sanger sequenced. Indicated is the isolate, which gene was mutated, and whether this resulted in nonsynonymous or frameshifts that affected the coding sequence. We observed HXK2 mutations frequently in short-lagged strains (Dataset S2) and STD1 mutations only once.
(XLSX)

Dataset S4 **Overview of primers and strains used.** This dataset relates to every figure in the article. All strains indicated here, and a description of their genotype is provided. Primers' usage is in Text S1.
(XLSX)

Dataset S5 **Overview of experimental details for every figure.** This dataset relates to every figure in the article. Here we include very precise experimental protocols to help people in the future to reproduce the results we have reported in this article. Lag phases and heterogeneous single-cell clonal behavior are technically challenging to measure reproducibly. Every figure has an in-depth explanation of how the data were acquired. Where experiments were exactly the same, we refer to the first figure where this information was provided.
(XLSX)

Dataset S6 **Cell density versus Bioscreen OD600 values.** Correlation of cell density with Bioscreen C plate reader's optical density measurements. Late log-phase AN296 cells were diluted to varying degrees to determine the linear range of the spectrophotometer. Reported in this table are values that fall within that linear range; higher cell densities are less sensitive to cell concentration, and lower densities cannot be reliably measured.
(XLSX)

Dataset S7 **Example of how growth rates were analyzed.** Here we provide an overview of how raw plate reader values from the Bioscreen C plate reader were analyzed (for S288c strain AN63). We illustrate in MS Excel how OD versus specific growth rate could be plotted using raw time versus OD readings and how noisy these measurements are across the early parts of the growth curve at low OD values (when growth rates are maximal). Further to the right, we show how the raw values and calculations relate to the output generated by our custom analysis scripts when R's smooth.spline (with spar = 0.35) function is first used on the data for smoothing, and finally how the script output can be used to plot multiple samples′ growth rates.
(XLSX)

Dataset S8 **Parameters used in the model reported in** **Figure 7** **.**
(XLSX)

Figure S1 (A) The lag phase when cells transition from the preferred carbon source glucose to another carbon source (diauxic shift) is highly variable between strains and is characterized by three phases: first, deceleration from a MaxR as glucose is depleted; second, a local minimum; and finally reacceleration to adapted growth on the alternative carbon source. The figure shows the growth profiles of four selected yeast strains that illustrate how the lag characteristics vary depending on the strain and on the alternative carbon source provided. The orange line plots the instantaneous growth rates of strains in LG conditions to illustrate the three characteristic phases of the lag phase. The purple and blue lines represent the log2 of the fold growth rate increase of the culture with LG + maltose or LG + galactose relative to LG alone. The finely dotted line corresponds to a relative log2 growth rate equal to zero, where the culture's growth rate in LG maltose or galactose equals that of the culture growing in LG alone. These results illustrate how variable the response to diauxic shift can be across conditions and strains. Furthermore, it is very difficult to determine where the lag phase begins and ends. We therefore use the GMR to estimate the total fitness of the strain across the growth curve, as well as single-cell measurements of the lag phase (see main text and Figure 1). (B) Different yeast strains show different variability in fitness (GMR) across different growth media. For each strain, we plot the ratio of its fitness (GMR) in stable glucose (HG) over its fitness in variable conditions (LG, LG + maltose, LG + galactose) so that higher values represent a larger drop in fitness in the mixed media compare to stable glucose. The strains are sorted according to the variability in GMR values. Cultures growing in abundant glucose conditions have GMRs that are closer to their MaxR because the culture grows in glucose for a longer period of time. In LG media, where cultures must switch to alternative carbon sources, the GMR is considerably lower than the MaxR. Cultures are generally able to maintain more steady rates of growth in the transition to maltose, followed by galactose. For all cultures, the LG condition leads to the most variable growth rates. However, some strains are able to maintain relatively similar fitness (GMR) in stable (HG) and variable (LG, LG + maltose, LG + galactose) media, whereas others show very variable fitness values. To summarize how a strain's fitness is affected by the carbon source, we defined a single metric that represents the “fitness variability,” calculated as the relative GMR in HG conditions divided by the geometric mean of relative GMRs in the LG, LG + maltose, and LG + galactose conditions. (C–F) Global properties of the single-cell lag phase caused by sudden transfer from glucose to maltose of *S. cerevisiae*. Included here are many S288c and derivative clones, as well as the other strains reported in Figure 1. The data represent some 25,000 single-cell measurements across more than 100 experiments. (C) In general, populations with average lag phases longer than 10 h tended also to have a lower fraction of the population being able to resume growth after the lag phase, suggesting that some strains have difficulties surviving sudden nutrient transitions. (D) The minimum lag phase was positively correlated with the average lag phase, however many experiments led to cells with long average lag phases despite rather short minimal lag phases. (E) The standard deviation of single-cell lag phases is positively correlated with the average lag phase, implying that cells with longer lag phases have more heterogeneous growth patterns. (F) The median “noise” of single-cell lag times, measured by the standard deviation divided by the average lag phase, is about 0.3, with a slight right-handed tail corresponding to samples with especially heterogeneous lag phase durations.
(TIF)

Figure S2 **Escape from the lag phase depends on activation of genes required for the metabolism of alternative carbon sources.** (A) The lag phase becomes longer with increasing periods of pregrowth in glucose. Reference strain S288c (containing *MALS*-YeCitrine gene fusions to monitor *MAL*S gene activity) was first pregrown in maltose medium, and then transferred to glucose medium. Samples of the culture growing in glucose were taken at regular time intervals to measure the MalS concentrations (YeCitrine fluorescence intensities) and to determine the average lag phase of cells that were transferred back into maltose. The results show that cells that had been pregrown for longer times in glucose showed lower maltase concentrations and had longer average lag phases when they were transferred back to maltose. For the 0 h time point, we report the mean doubling-time of mother cells for samples coming directly from maltose (with no growth period in glucose at all). Error bars represent the standard deviation of ∼150 single cells measured. (B) Combined overexpression of genes encoding maltose permease (*MALT*) and maltase (*MAL*S) is sufficient to overcome severe glucose-to-maltose lag phase of the reference strain S288c. Single-cell lags of a wild-type reference strain or transformants of this strain engineered with GPD promoters overexpressing *MALT*, *MAL*S, or both were measured after 24 h of logarithmic growth in glucose. (C) Determination of the lag phase of individual cells in a population using time-lapse microscopy. The growth of several (typically 100–250) single cells that are transferred from one nutrient to another was followed using an automated time-lapse microscope with autofocus function (see Materials and Methods section for details). The resulting time-lapse frames were analyzed to determine the timing of the first budding event (i.e., the end of the lag phase). Shown are just a few images of a typical time lapse series of a maltose transporter *MALT*-YeCitrine–labeled strain. Note the correlation between the first budding event (i.e., the end of the lag phase) and the activation of the *MAL* genes (as measured by the YeCitrine fluorescent signal). (D) Distribution of the MalS-YeCitrine fluorescence intensities for the data reported in Figure 2. About 50% of the cells show fluorescence intensities above the background level of about 1800 A.U., indicative of MAL gene activation. (E) Different fluorescence cutoff values do not influence the conclusions reported in Figure 2. The plot relates to a statistical test of the data shown in Figure 2. It shows the *p* values of a Mann–Whitney U test for different cutoff values in fluorescence levels defining “maltose commitment,” showing that the result that “ON” cells grow more slowly for cutoff values above the background ∼1800 A.U. fluorescence units.
(TIF)

Figure S3 **Characterization of mutants isolated after repeated cycling of a strain with a long lag phase between glucose and maltose medium.** (A) Populations measured after the sixth cycle of growth in glucose and maltose show different lag behaviors. Depicted here are single-cell lag profiles from evolving populations that illustrate how various populations began to show shorter glucose-to-maltose lag phases compared to the ancestral strain S288c. This measurement was conducted after the end of the selection after the 12 independently evolving cultures had grown for 22 h in glucose. Genetically heterogeneous populations were washed into maltose-containing media as per the selection protocol, and spotted onto the agar pad for microscopic monitoring of lag phases. We isolated clones from four populations (numbers 2, 3, 6, and 10) to obtain Isolates 1, 2, 3, and 4 (shown in Figure 3). All populations except population 7 had significantly higher survival compared to the ancestor (logrank Chi-square >3.5, 1 df, *p*<0.05). (B) Lag phase duration of genetically heterogeneous evolving populations descending from the *MALT*- and *MAL*S-fluorescently labeled strains at different numbers of glucose–maltose cycles. Reported are the cumulative distribution histograms of single-cell lags of populations after the indicated rounds of glucose to maltose selection. The sixth population yielded clones with shorter lags and also strong flocculation characteristics, including isolate number 5 in Figure 3A. (C) Isolates from the *MALS-YeCitrine* fluorescently labeled populations show variable lag duration, some very short and others longer than the ancestral phenotype. Samples were grown for 6 h in glucose before transfer to maltose.
(TIF)

Figure S4 **Overview of growth parameters for evolved populations.** Overview of GMR (A) and MaxR (B) in either stable 3% glucose (HG), or environments where the cells need to shift between carbon sources (0.5% glucose alone (LG), or LG supplemented with either galactose or maltose). Shown are growth measurements of three random isolates from each of the 12 evolved populations. Note that several isolates show higher GMR (fitness), but lower MaxR in unstable environments that require a shift between glucose and a nonpreferred carbon source. (C) High fitness (GMR) in media requiring a switch from glucose to a nonpreferred carbon source comes at a fitness tradeoff of slower maximal growth speed in stable glucose conditions. Isolates show an inverse correlation between GMR and MaxR in variable environments (LG, LG + Mal, LG + Gal) and a positive correlation between GMR and MaxR in stable glucose (HG) conditions (in green). GMR and MaxR values are expressed relative to the growth rate of the ancestral strain measured in the same conditions. Error bars represent the standard deviation of 2–4 biological replicates. (D) Isolates that show shorter lag phases also show reduced variation in fitness (GMR) across changing environments. As in Figure 1H we computed the normalized (GMR/MaxR) for HG conditions relative to the geometric mean of normalized GMR in variable environments (LG, LG + galactose, and LG + maltose). The vertical axis shows the average single-cell lag phase of cells in maltose after 6 h of growth in glucose. The black circles are the averages of these values for the three isolates from each population, with error bars equal to the maximal and minimal value observed for isolates within the population. The red triangle represents the ancestral strain, and the grey circles are the same data as reported in Figure 1H.
(TIF)

Figure S5 **Mutants isolated after repeated cycling between glucose and maltose of a strain with a long lag phase show altered** ***MAL*** **gene expression patterns and hysteresis.** (A) *MAL*S gene expression in glucose correlates with growth measurements described in Figure 4. Specifically, strains with high MALS background expression in glucose show higher fitness across variable environments, shorter lag phases upon transfer to maltose, and lower MaxR's. Correlations excluded flocculent strains. (B) To demonstrate variability and epigenetic inheritance of *MAL* expression states, a culture of Isolate 1 bearing a MALS-mCherry allele pregrown in glucose was grown exponentially in mixed glucose + maltose medium for 24 h, and then transferred to the same maltose + glucose medium for time-lapse microscopy. Cell growth was monitored by brightfield microscopy and *MALS* expression by MalS-mCherry fluorescence signal. (C) Similarly to (B), *MALS* gene expression (through *MALS-mCherry* gene fusions) was tracked during growth for the ancestral strain as well as Isolate 1 coming from a different precondition. Cells in microcolonies of ancestral cells do not express their *MAL* genes in the glucose–maltose medium. A fluorescent particle is included in this image to show that there was signal detection. On the other hand, a microcolony of evolved cells (Isolate 1) that did not show any *MAL* expression when it was inoculated showed slow stochastic activation of *MAL* genes in some cells as the microcolony grew. (D) The *MAL* activator is necessary for high levels of *MAL* gene expression in maltose–glucose media. Each pair of blue and red violin plots represents gene expression data for individual cells determined by microscopy for various derivatives of Isolate 1, an *HXK2* mutant. The leftmost pair are plots of Isolate 1 bearing a *MALS-mCherry* construct; the middle pair is the same strain with the *MAL63* activator gene deleted, and the right-most pair is Isolate 1 without the *MALS-mCherry* gene. Prior to determination of fluorescence intensities, all samples were growing exponentially at low cell densities in either maltose–glucose media or glucose media of the same osmolarity (containing sorbitol) and measurements represent ∼150 cells per genotype–condition combination. A significant effect for genotype (F = 47, df = 2, *p* = 0) and media (F = 159, df = 1, *p* = 0) as well as a Genotype×Media interaction (F = 46, df = 2, *p* = 0) were detected by ANOVA analysis. Comparisons using Mann–Whitney U tests suggest that leaky expression of the *MALS* gene in glucose-alone media is not dependent upon the *MAL63* activator (*p*<1×2^−8^, Bonferroni-corrected *p* value) but that the activator is required for the high levels of *MALS* expression in maltose–glucose media (*p* = 0).
(TIF)

Figure S6 **Dynamics of** ***MAL*** **gene regulation of four** ***HXK2*** **mutants compared to the ancestral strain.** The regulation of *MAL* gene expression is highly variable across evolved *HXK2* alleles. Shown are flow cytometry histograms of four *MALS-mCherry/MALT-YeCitrine* labeled isolates and the ancestral clone growing in the presence of abundant concentrations of maltose and glucose, coming from two initial conditions: *MAL* induced (ON; pre-growth in maltose-containing media) and *MAL* uninduced (OFF), where cells were pregrown in glucose-containing media. Samples were grown at low cell densities for approximately seven generations. Note the wide variability in response of the *MAL* genes of these different *HXK2* mutants to the maltose + glucose media compared to the ancestral clone. In particular, Isolate 8.1 represents an extreme case of *HXK2* inactivation, where due to an AUG > GUG mutation at the start codon of *HXK2* this mutant likely has very little Hxk2p expression. This isolate maintains high levels of expression when it is coming from maltose, and likewise induces the *MAL* genes quickly when maltose is added to glucose medium. At the other extreme, Isolate 11.1 has a slow rate of OFF to ON switching and represses the *MAL* genes almost as tightly as the ancestral clone. These data are quantified in Figure S7.
(TIF)

Figure S7 **Mal protein is produced at different rates in** ***HXK2*** **mutants.** The data reported in Figure S6 were analyzed at the population level using growth and gene expression data to determine differences in gene regulation between different evolved isolates. (A) Different evolved isolates show different average MalS expression patterns in glucose + maltose mixtures (each data point represents one biological replicate). (B) Some populations show (partial) loss of glucose repression and actively produce new MalS proteins in glucose + maltose medium. The grey dotted line shows the expected MalS signal for a culture that originally has its MAL genes ON and then turned them off during growth. As expected, the ancestral strain follows the predicted pattern, indicating silencing of MalS expression in the presence of glucose. However, Isolate pop8.1 produces MalS protein at a much higher rate than would be expected according to this null model. (C) The *HXK2* mutant isolates (*n* = 2 per strain and condition) produce higher levels of MalS protein when the culture originally had its MAL genes on (blue diamonds) compared to cultures that began growth in maltose–glucose media with their MAL genes OFF (red circles). Values are the MalS protein produced per generation over the final two time points (depicted in A). Two-way ANOVA analysis of protein production rate as a function of genotype or original media indicates a significant effect for genotype (F = 162.18, df = 4, *p*<0.001), original media (glucose or maltose; F = 108.31, df = 1, *p*<0.001), and a Genotype×Original Media interaction (F = 27.74, df = 4, *p*<0.001).
(TIF)

Figure S8 **Mal protein is produced at different rates in** ***HXK2*** **mutants.** (A) Population-level *MAL* gene expression is correlated with population-level growth rate hysteresis in mixed media. Each dot corresponds to one isolate pregrown exponentially either in maltose (diamonds) or glucose (circles), then transferred as exponentially growing cultures to a mixture of maltose and glucose. Growth rates were measured in a plate reader within 8 h of inoculation. Plotted along the horizontal axis is the average MalS-mCherry fluorescence signal (Dataset S2). Error bars represent the standard deviation of at least two biological replicates. (B) The *MAL* activator causes slow growth rates in maltose–glucose media. Shown are the mean growth rates of *n* = 3 measurements of Isolate 1 with and without the *MAL* activator *MAL63*, growing in maltose–glucose or glucose media alone. ANOVA analysis shows a significant effect for genotype (F = 12.47, df = 2, *p*<0.01), growth media condition (F = 23.43, df = 1, *p*<0.01), and Genotype×Media interaction (F = 15.48, df = 2, *p*<0.01). The *t* tests between strains and conditions indicate that the strain with the *MAL*-activator grows significantly more slowly in maltose–glucose media than in glucose media alone (two-tailed *t* test, *p*<0.04 with Bonferroni correction). (C) Induction of *MAL* genes in glucose medium comes at a fitness cost in single cells. Each dot represents the correlation between specific growth rate of microcolonies and their final MalS-mCherry expression state. These data were analyzed from the experiment depicted in Figure S5B and C and as described in Materials and Methods and Dataset S5. The final expression state of the microcolony is anitcorrelated with its growth rate, demonstrating that induction of *MAL* genes in medium containing glucose comes at a fitness cost. (D and E) Activation of the *MAL* genes in glucose + maltose medium increases fitness upon transfer to maltose medium. An evolved clone bearing the *MALS-mCherry* reporter construct (Isolate 1, an *HXK2* mutant) was transferred from maltose + glucose mixture to maltose-only media. Initial fluorescence intensity of the cells was recorded with a single image, followed by single-cell lag times via brightfield time-lapse microscopy. In (F), each circle represents a single cell's lag phase and initial fluorescence intensity. In (G) we show cumulative distribution histograms between ON and OFF samples to illustrate the fitness gained by induction of the *MAL* genes (samples greater than or less than 1,000 A.U. fluorescence units, respectively).
(TIF)

Movie S1 **S288c (strain AN148) tagged with** ***MAL11-YeCitrine*** **was grown in glucose for 6 h and then transferred to maltose media.** Microscopic images were acquired every 30 min in brightfield (DIC) and YFP fluorescence channel settings. Note the heterogeneity in lag phase duration between isogenic cells and how the end of each cell's lag phase correlates with induction of the YeCitrine reporter.
(MP4)

Movie S2 **Strain UWOPS83-787.3 (strain in** **Figure 1** **with low fitness variability) was grown as above and growth monitored by brightfield time-lapse microscopy.** Note the shorter average lag phases and reduced heterogeneity in lag phase duration between isogenic cells compared to S288c.
(MP4)

Movie S3 **Strain AN296 (ancestral strain for the constitutively labeled YeCitrine strain) was grown for 22 h in glucose media and then transferred to maltose.** Time-lapse DIC imaging shows that the majority of cells do not survive this treatment.
(MP4)

Movie S4 **Evolved Isolate 1 was grown for 22 h in glucose media and transferred to maltose media for time-lapse microscopy (same as Movie S3).** Note that relative to the ancestor, this strain has significantly increased survival, shortened lag phase length and decreased intercellular heterogeneity.
(MP4)

Movie S5 **Same as above for Isolate 3.** Note that this strains' lag phase lengths are more heterogeneous but notably shorter with higher survival than the ancestor.
(MP4)

Movie S6 **Isolate 1 transformed with a** ***MALS-mCherry*** **construct is shown.** This movie shows the time-lapse microscopic data from the same cells depicted in Figure S5B and S5C. The sample's *MAL* genes were multimodally induced by pregrowth in maltose + glucose media (initial state uninduced) for 24 h before transferring to the maltose + glucose media for time-lapse microscopy. Note that induced and uninduced states propagate within microcolonies for many generations.
(MP4)

Movie S7 **This is the same strain as in Movie S6, depicted in Figure S5B and S5C.** This time the strain was pregrown in glucose-only media and therefore transferred to the maltose + glucose mixture with *MAL* genes uninduced. Note that as cells grow, their *MAL* genes slowly and stochastically switch on. These events propagate within lineages of individual microcolonies, often resulting in sectoring within microcolonies.
(MP4)

Movie S8 **The ancestral strain transformed with a *MALS-mCherry* construct was pregrown in maltose + glucose media as in Figure S5c and Movie S6.** Note that its *MAL* genes do not activate during the course of the experiment.
(MP4)

Text S1 **Supplementary Methods.**
(DOCX)

## Abbreviations

CNV: copy number variation
Gal: galactose
GMR: geometric mean growth rate
GO: gene ontological
HG: high glucose
LG: low glucose
Mal: maltose
MalR: maltose regulator
MalS: maltase
MalT: maltose transporter
MaxR: maximal growth rate
PKA: protein kinase A
