# Supplementary figures and images for: Different Levels of Catabolite Repression Optimize Growth in Stable and Variable Environments
Source: PLoS Biol. 2014 Jan 14;12(1):e1001764. doi: 10.1371/journal.pbio.1001764 (PMC3891604; doi:10.1371/journal.pbio.1001764)

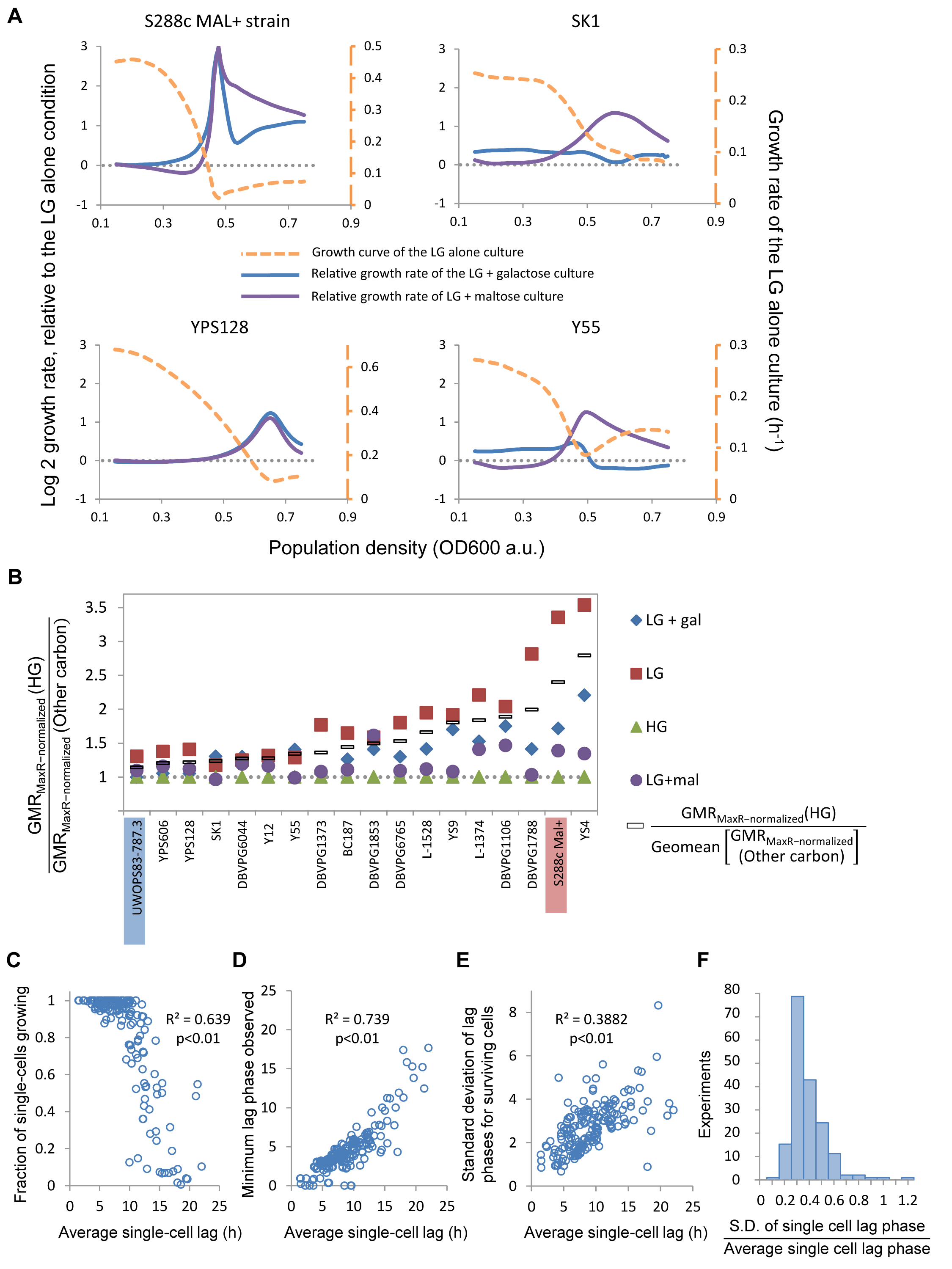

Supplement: Figure S1 — (A) The lag phase when cells transition from the preferred carbon source glucose to another carbon source (diauxic shift) is highly variable between strains and is characterized by three phases: first, deceleration from a MaxR as glucose is depleted; second, a local minimum; and finally reacceleration to adapted growth on the alternative carbon source. The figure shows the growth profiles of four selected yeast strains that illustrate how the lag characteristics vary depending on the strain and on the alternative carbon source provided. The orange line plots the instantaneous growth rates of strains in LG conditions to illustrate the three characteristic phases of the lag phase. The purple and blue lines represent the log2 of the fold growth rate increase of the culture with LG + maltose or LG + galactose relative to LG alone. The finely dotted line corresponds to a relative log2 growth rate equal to zero, where the culture's growth rate in LG maltose or galactose equals that of the culture growing in LG alone. These results illustrate how variable the response to diauxic shift can be across conditions and strains. Furthermore, it is very difficult to determine where the lag phase begins and ends. We therefore use the GMR to estimate the total fitness of the strain across the growth curve, as well as single-cell measurements of the lag phase (see main text and Figure 1). (B) Different yeast strains show different variability in fitness (GMR) across different growth media. For each strain, we plot the ratio of its fitness (GMR) in stable glucose (HG) over its fitness in variable conditions (LG, LG + maltose, LG + galactose) so that higher values represent a larger drop in fitness in the mixed media compare to stable glucose. The strains are sorted according to the variability in GMR values. Cultures growing in abundant glucose conditions have GMRs that are closer to their MaxR because the culture grows in glucose for a longer period of time. In LG media, where cultur [file pbio.1001764.s009.tif]

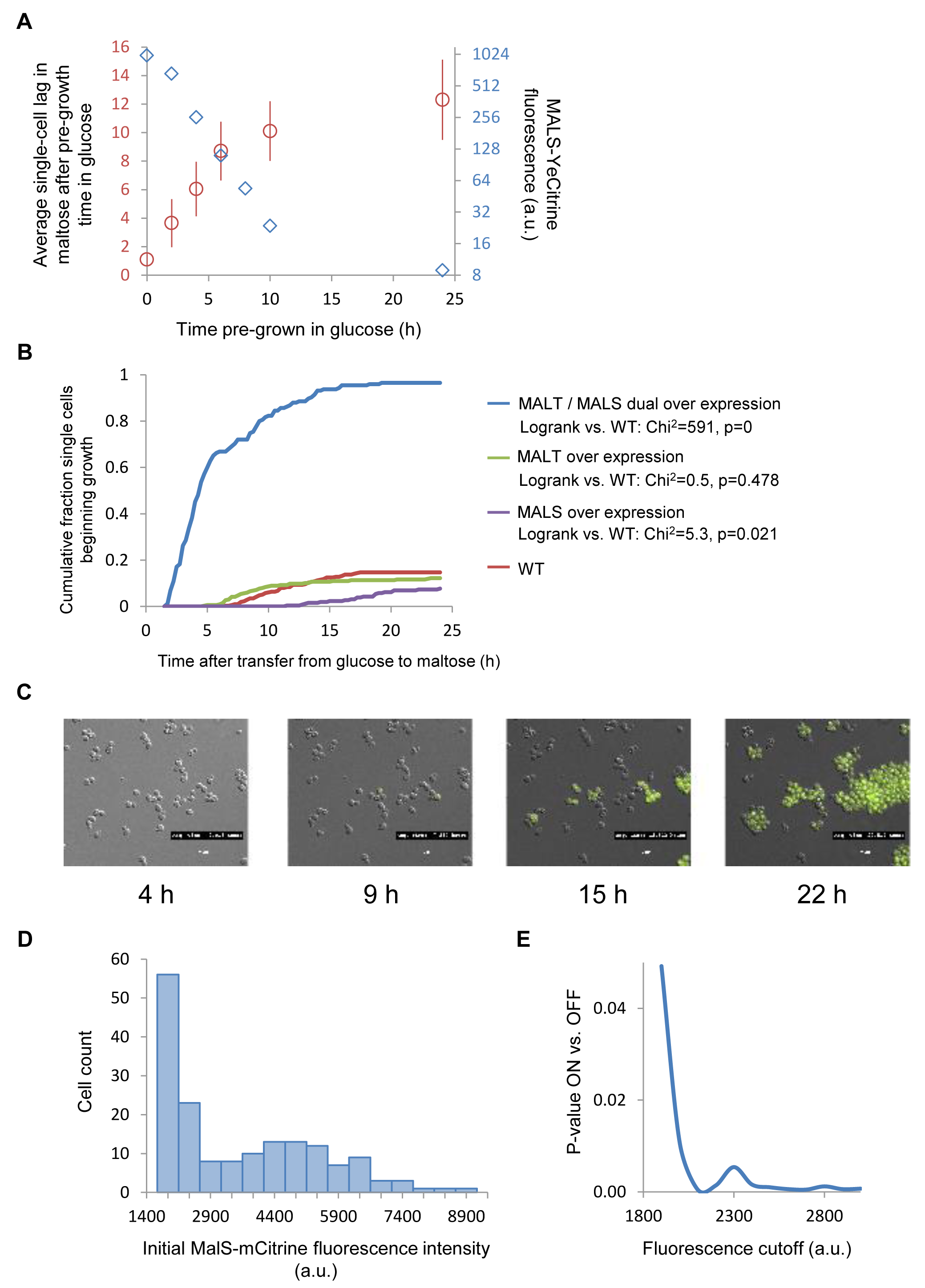

Supplement: Figure S2 — Escape from the lag phase depends on activation of genes required for the metabolism of alternative carbon sources. (A) The lag phase becomes longer with increasing periods of pregrowth in glucose. Reference strain S288c (containing MALS-YeCitrine gene fusions to monitor MALS gene activity) was first pregrown in maltose medium, and then transferred to glucose medium. Samples of the culture growing in glucose were taken at regular time intervals to measure the MalS concentrations (YeCitrine fluorescence intensities) and to determine the average lag phase of cells that were transferred back into maltose. The results show that cells that had been pregrown for longer times in glucose showed lower maltase concentrations and had longer average lag phases when they were transferred back to maltose. For the 0 h time point, we report the mean doubling-time of mother cells for samples coming directly from maltose (with no growth period in glucose at all). Error bars represent the standard deviation of ∼150 single cells measured. (B) Combined overexpression of genes encoding maltose permease (MALT) and maltase (MALS) is sufficient to overcome severe glucose-to-maltose lag phase of the reference strain S288c. Single-cell lags of a wild-type reference strain or transformants of this strain engineered with GPD promoters overexpressing MALT, MALS, or both were measured after 24 h of logarithmic growth in glucose. (C) Determination of the lag phase of individual cells in a population using time-lapse microscopy. The growth of several (typically 100–250) single cells that are transferred from one nutrient to another was followed using an automated time-lapse microscope with autofocus function (see Materials and Methods section for details). The resulting time-lapse frames were analyzed to determine the timing of the first budding event (i.e., the end of the lag phase). Shown are just a few images of a typical time lapse series of a maltose transporter MALT-YeCitrine–labeled strain. [file pbio.1001764.s010.tif]

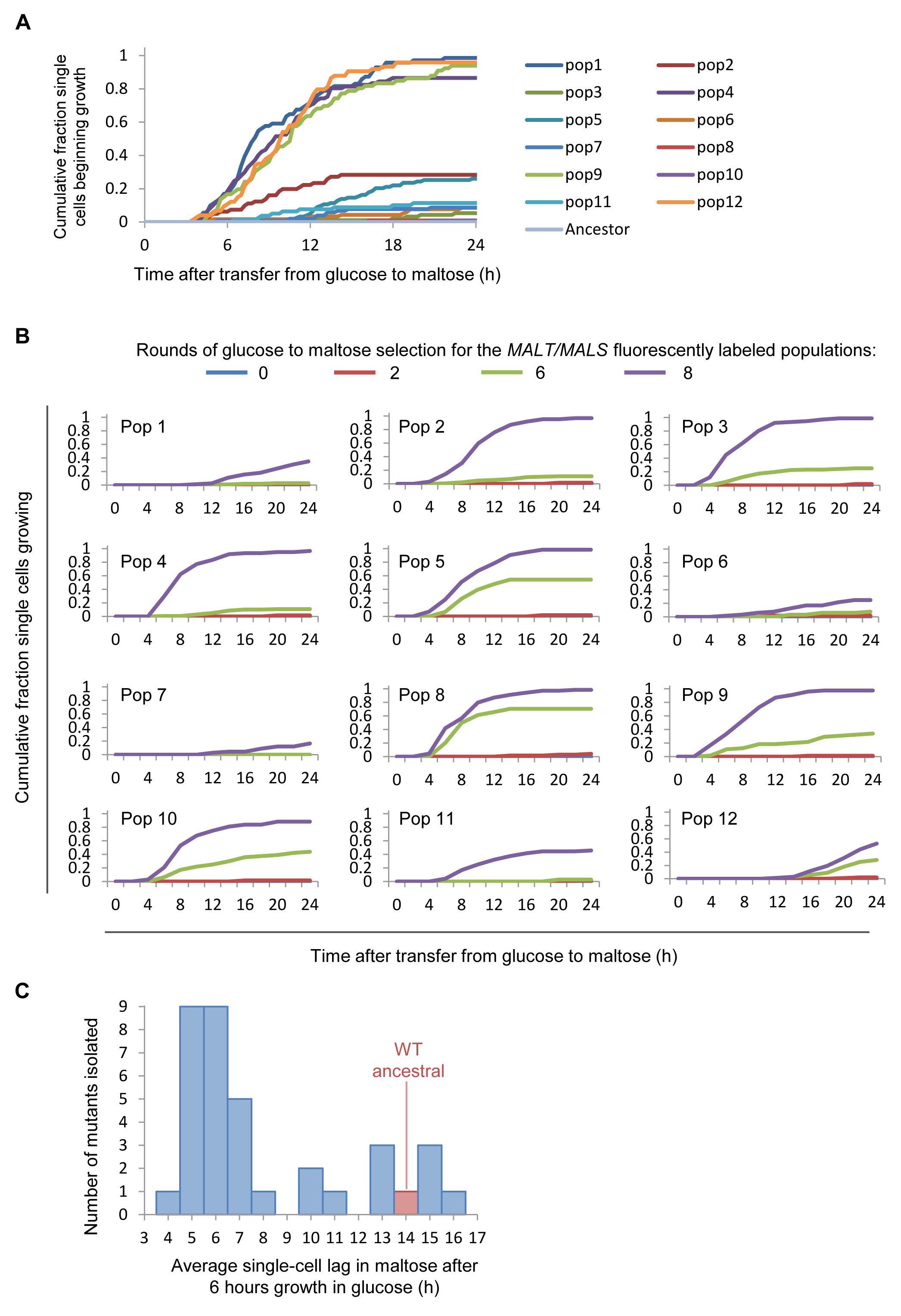

Supplement: Figure S3 — Characterization of mutants isolated after repeated cycling of a strain with a long lag phase between glucose and maltose medium. (A) Populations measured after the sixth cycle of growth in glucose and maltose show different lag behaviors. Depicted here are single-cell lag profiles from evolving populations that illustrate how various populations began to show shorter glucose-to-maltose lag phases compared to the ancestral strain S288c. This measurement was conducted after the end of the selection after the 12 independently evolving cultures had grown for 22 h in glucose. Genetically heterogeneous populations were washed into maltose-containing media as per the selection protocol, and spotted onto the agar pad for microscopic monitoring of lag phases. We isolated clones from four populations (numbers 2, 3, 6, and 10) to obtain Isolates 1, 2, 3, and 4 (shown in Figure 3). All populations except population 7 had significantly higher survival compared to the ancestor (logrank Chi-square >3.5, 1 df, p<0.05). (B) Lag phase duration of genetically heterogeneous evolving populations descending from the MALT- and MALS-fluorescently labeled strains at different numbers of glucose–maltose cycles. Reported are the cumulative distribution histograms of single-cell lags of populations after the indicated rounds of glucose to maltose selection. The sixth population yielded clones with shorter lags and also strong flocculation characteristics, including isolate number 5 in Figure 3A. (C) Isolates from the MALS-YeCitrine fluorescently labeled populations show variable lag duration, some very short and others longer than the ancestral phenotype. Samples were grown for 6 h in glucose before transfer to maltose. (TIF) [file pbio.1001764.s011.tif]

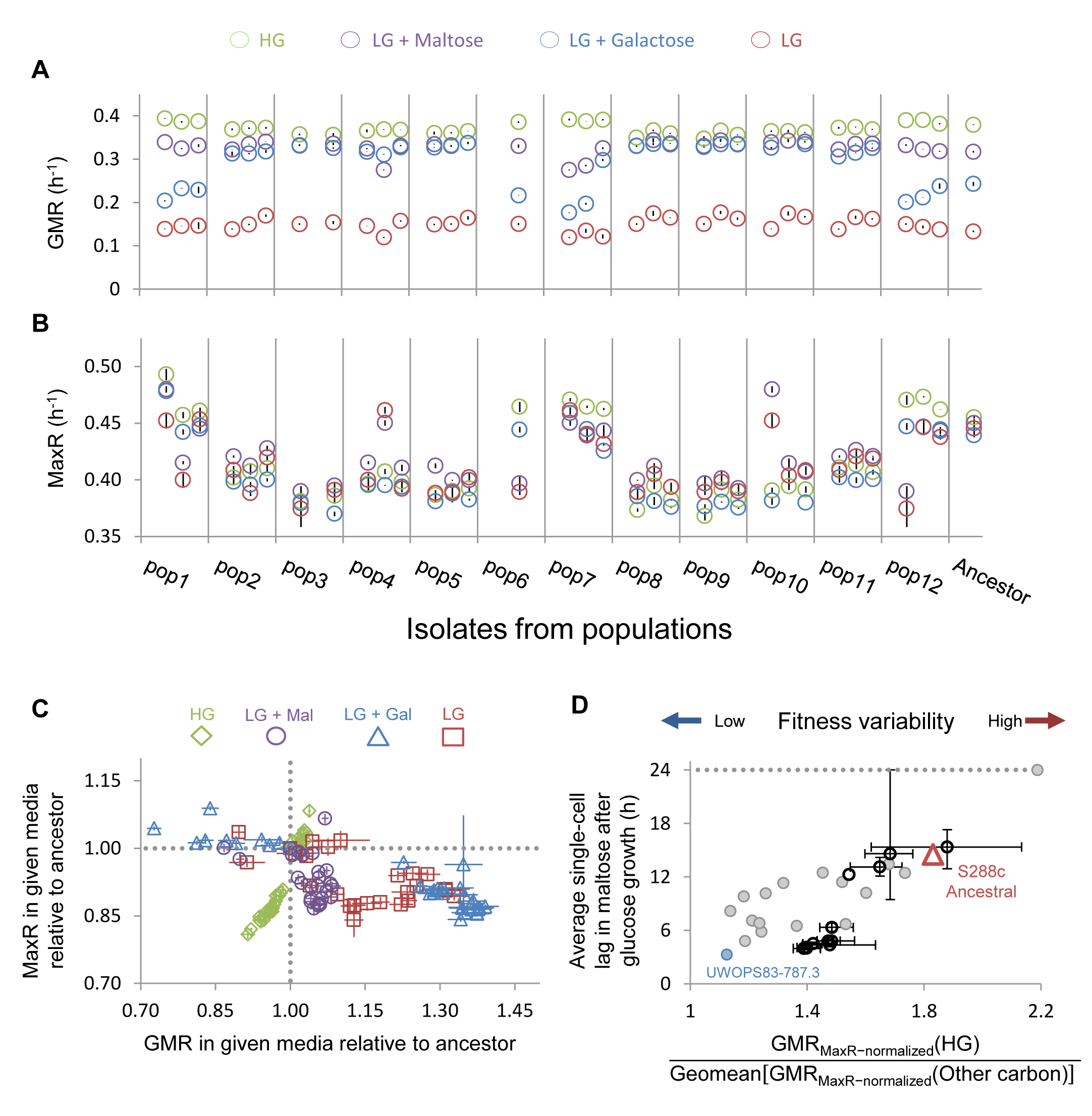

Supplement: Figure S4 — Overview of growth parameters for evolved populations. Overview of GMR (A) and MaxR (B) in either stable 3% glucose (HG), or environments where the cells need to shift between carbon sources (0.5% glucose alone (LG), or LG supplemented with either galactose or maltose). Shown are growth measurements of three random isolates from each of the 12 evolved populations. Note that several isolates show higher GMR (fitness), but lower MaxR in unstable environments that require a shift between glucose and a nonpreferred carbon source. (C) High fitness (GMR) in media requiring a switch from glucose to a nonpreferred carbon source comes at a fitness tradeoff of slower maximal growth speed in stable glucose conditions. Isolates show an inverse correlation between GMR and MaxR in variable environments (LG, LG + Mal, LG + Gal) and a positive correlation between GMR and MaxR in stable glucose (HG) conditions (in green). GMR and MaxR values are expressed relative to the growth rate of the ancestral strain measured in the same conditions. Error bars represent the standard deviation of 2–4 biological replicates. (D) Isolates that show shorter lag phases also show reduced variation in fitness (GMR) across changing environments. As in Figure 1H we computed the normalized (GMR/MaxR) for HG conditions relative to the geometric mean of normalized GMR in variable environments (LG, LG + galactose, and LG + maltose). The vertical axis shows the average single-cell lag phase of cells in maltose after 6 h of growth in glucose. The black circles are the averages of these values for the three isolates from each population, with error bars equal to the maximal and minimal value observed for isolates within the population. The red triangle represents the ancestral strain, and the grey circles are the same data as reported in Figure 1H. (TIF) [file pbio.1001764.s012.tif]

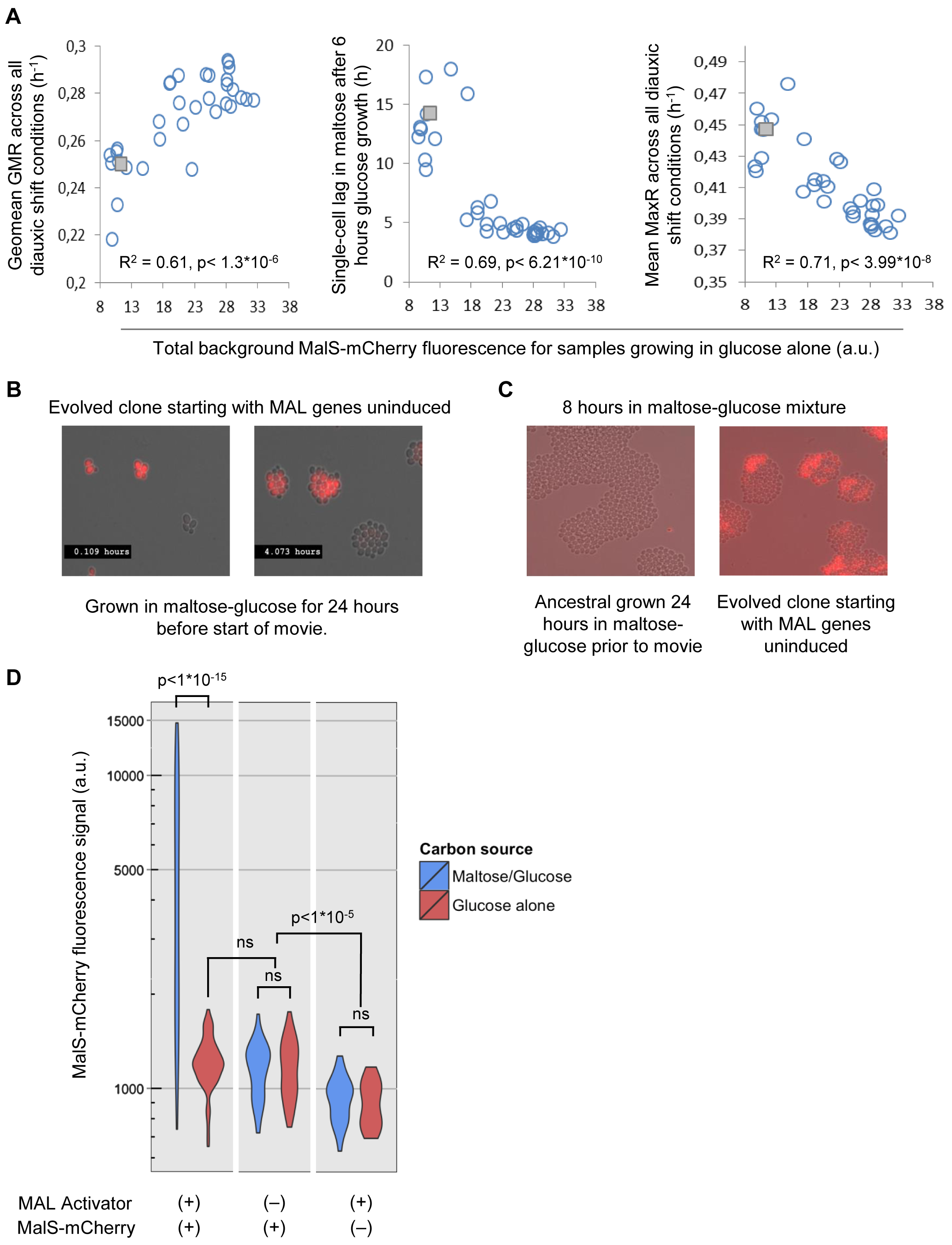

Supplement: Figure S5 — Mutants isolated after repeated cycling between glucose and maltose of a strain with a long lag phase show altered MAL gene expression patterns and hysteresis. (A) MALS gene expression in glucose correlates with growth measurements described in Figure 4. Specifically, strains with high MALS background expression in glucose show higher fitness across variable environments, shorter lag phases upon transfer to maltose, and lower MaxR's. Correlations excluded flocculent strains. (B) To demonstrate variability and epigenetic inheritance of MAL expression states, a culture of Isolate 1 bearing a MALS-mCherry allele pregrown in glucose was grown exponentially in mixed glucose + maltose medium for 24 h, and then transferred to the same maltose + glucose medium for time-lapse microscopy. Cell growth was monitored by brightfield microscopy and MALS expression by MalS-mCherry fluorescence signal. (C) Similarly to (B), MALS gene expression (through MALS-mCherry gene fusions) was tracked during growth for the ancestral strain as well as Isolate 1 coming from a different precondition. Cells in microcolonies of ancestral cells do not express their MAL genes in the glucose–maltose medium. A fluorescent particle is included in this image to show that there was signal detection. On the other hand, a microcolony of evolved cells (Isolate 1) that did not show any MAL expression when it was inoculated showed slow stochastic activation of MAL genes in some cells as the microcolony grew. (D) The MAL activator is necessary for high levels of MAL gene expression in maltose–glucose media. Each pair of blue and red violin plots represents gene expression data for individual cells determined by microscopy for various derivatives of Isolate 1, an HXK2 mutant. The leftmost pair are plots of Isolate 1 bearing a MALS-mCherry construct; the middle pair is the same strain with the MAL63 activator gene deleted, and the right-most pair is Isolate 1 without the MALS-mCherry gene. Prior to determination [file pbio.1001764.s013.tif]

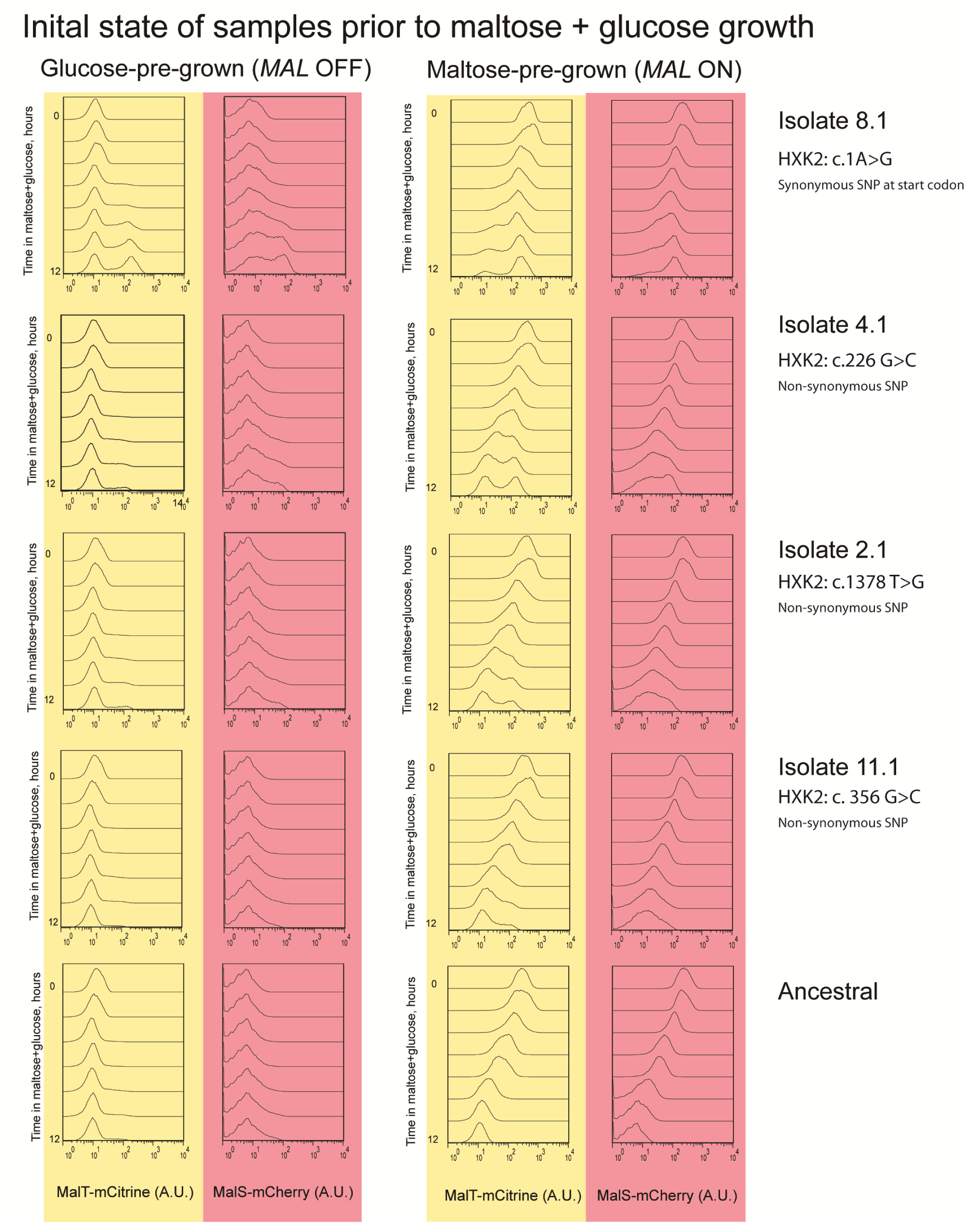

Supplement: Figure S6 — Dynamics of MAL gene regulation of four HXK2 mutants compared to the ancestral strain. The regulation of MAL gene expression is highly variable across evolved HXK2 alleles. Shown are flow cytometry histograms of four MALS-mCherry/MALT-YeCitrine labeled isolates and the ancestral clone growing in the presence of abundant concentrations of maltose and glucose, coming from two initial conditions: MAL induced (ON; pre-growth in maltose-containing media) and MAL uninduced (OFF), where cells were pregrown in glucose-containing media. Samples were grown at low cell densities for approximately seven generations. Note the wide variability in response of the MAL genes of these different HXK2 mutants to the maltose + glucose media compared to the ancestral clone. In particular, Isolate 8.1 represents an extreme case of HXK2 inactivation, where due to an AUG > GUG mutation at the start codon of HXK2 this mutant likely has very little Hxk2p expression. This isolate maintains high levels of expression when it is coming from maltose, and likewise induces the MAL genes quickly when maltose is added to glucose medium. At the other extreme, Isolate 11.1 has a slow rate of OFF to ON switching and represses the MAL genes almost as tightly as the ancestral clone. These data are quantified in Figure S7. (TIF) [file pbio.1001764.s014.tif]

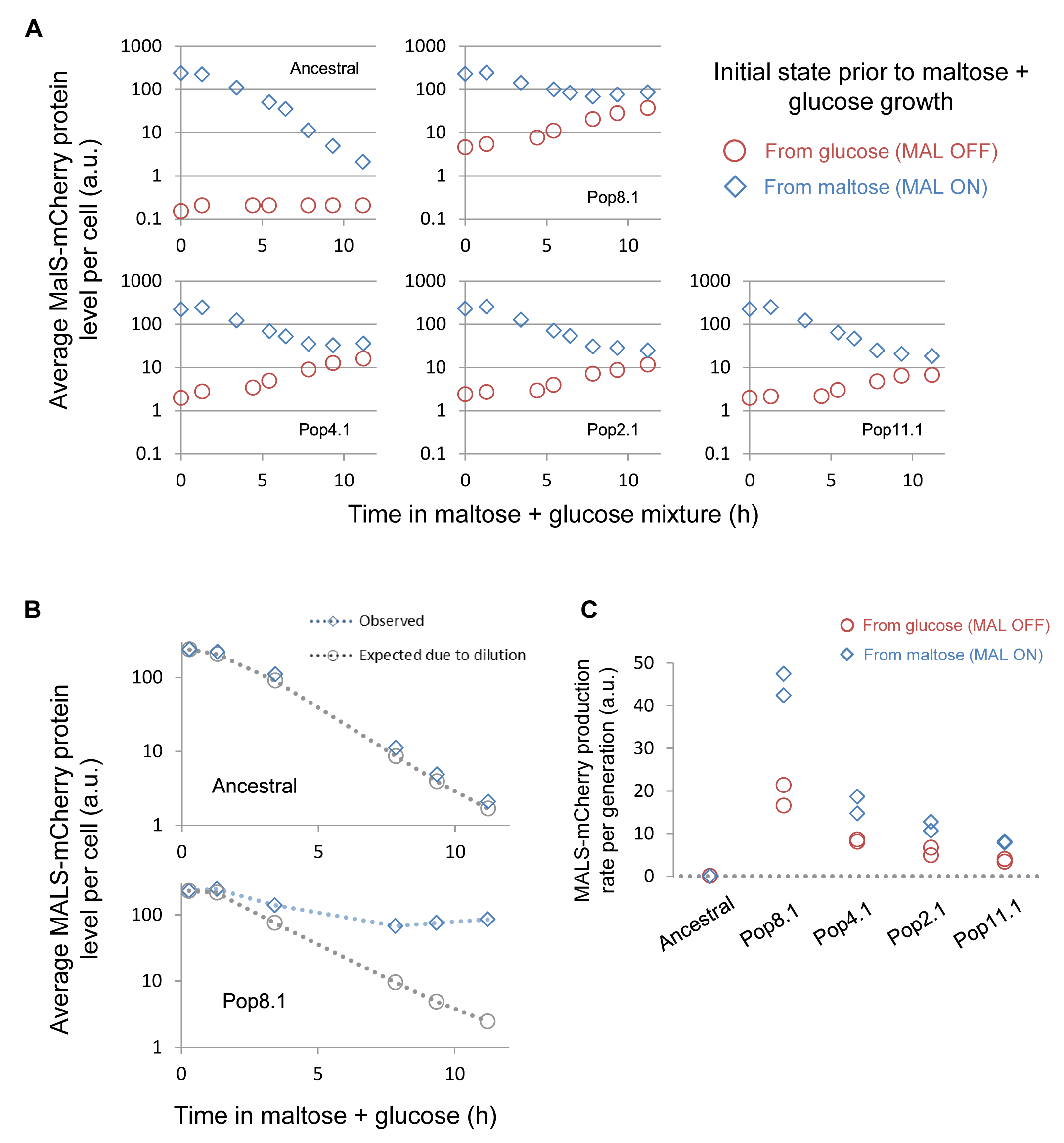

Supplement: Figure S7 — Mal protein is produced at different rates in HXK2 mutants. The data reported in Figure S6 were analyzed at the population level using growth and gene expression data to determine differences in gene regulation between different evolved isolates. (A) Different evolved isolates show different average MalS expression patterns in glucose + maltose mixtures (each data point represents one biological replicate). (B) Some populations show (partial) loss of glucose repression and actively produce new MalS proteins in glucose + maltose medium. The grey dotted line shows the expected MalS signal for a culture that originally has its MAL genes ON and then turned them off during growth. As expected, the ancestral strain follows the predicted pattern, indicating silencing of MalS expression in the presence of glucose. However, Isolate pop8.1 produces MalS protein at a much higher rate than would be expected according to this null model. (C) The HXK2 mutant isolates (n = 2 per strain and condition) produce higher levels of MalS protein when the culture originally had its MAL genes on (blue diamonds) compared to cultures that began growth in maltose–glucose media with their MAL genes OFF (red circles). Values are the MalS protein produced per generation over the final two time points (depicted in A). Two-way ANOVA analysis of protein production rate as a function of genotype or original media indicates a significant effect for genotype (F = 162.18, df = 4, p<0.001), original media (glucose or maltose; F = 108.31, df = 1, p<0.001), and a Genotype×Original Media interaction (F = 27.74, df = 4, p<0.001). (TIF) [file pbio.1001764.s015.tif]

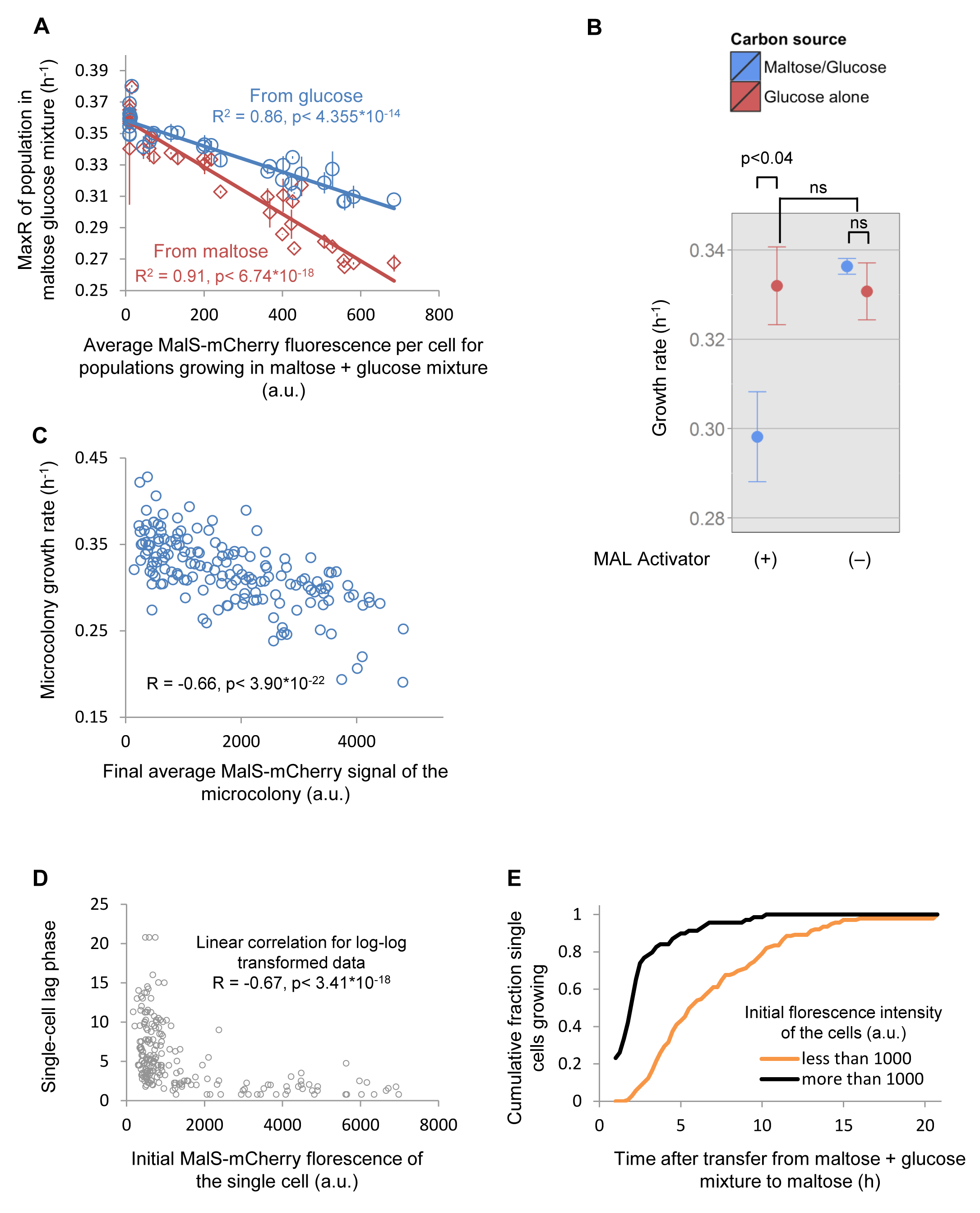

Supplement: Figure S8 — Mal protein is produced at different rates in HXK2 mutants. (A) Population-level MAL gene expression is correlated with population-level growth rate hysteresis in mixed media. Each dot corresponds to one isolate pregrown exponentially either in maltose (diamonds) or glucose (circles), then transferred as exponentially growing cultures to a mixture of maltose and glucose. Growth rates were measured in a plate reader within 8 h of inoculation. Plotted along the horizontal axis is the average MalS-mCherry fluorescence signal (Dataset S2). Error bars represent the standard deviation of at least two biological replicates. (B) The MAL activator causes slow growth rates in maltose–glucose media. Shown are the mean growth rates of n = 3 measurements of Isolate 1 with and without the MAL activator MAL63, growing in maltose–glucose or glucose media alone. ANOVA analysis shows a significant effect for genotype (F = 12.47, df = 2, p<0.01), growth media condition (F = 23.43, df = 1, p<0.01), and Genotype×Media interaction (F = 15.48, df = 2, p<0.01). The t tests between strains and conditions indicate that the strain with the MAL-activator grows significantly more slowly in maltose–glucose media than in glucose media alone (two-tailed t test, p<0.04 with Bonferroni correction). (C) Induction of MAL genes in glucose medium comes at a fitness cost in single cells. Each dot represents the correlation between specific growth rate of microcolonies and their final MalS-mCherry expression state. These data were analyzed from the experiment depicted in Figure S5B and C and as described in Materials and Methods and Dataset S5. The final expression state of the microcolony is anitcorrelated with its growth rate, demonstrating that induction of MAL genes in medium containing glucose comes at a fitness cost. (D and E) Activation of the MAL genes in glucose + maltose medium increases fitness upon transfer to maltose medium. An evolved clone bearing the MALS-mCherry reporter construct (Isolat [file pbio.1001764.s016.tif]
