# Supplementary material for: Different Levels of Catabolite Repression Optimize Growth in Stable and Variable Environments
Source: PLoS Biol. 2014 Jan 14;12(1):e1001764. doi: 10.1371/journal.pbio.1001764 (PMC3891604; doi:10.1371/journal.pbio.1001764)
Supplement: Text S1 — Supplementary Methods. (DOCX) [file pbio.1001764.s025.docx]

*Text S1*

Precise experimental details for every experiment reported in the manuscript and figures can be found in dataset S5.

*Strain propagation and genetic construction*

All strains were stored long-term at -80 in 25% glycerol. Standard complex undefined growth media containing 10 g yeast extract + 20 g peptone per 1 L distilled water (YP media, BD Bacto Peptone REF#211677) [1] was used for propagation and experiments reported, except those involving fluorescence microscopy, where SC media was used (see experimental details Dataset S5 [1]). Filter-sterilized maltose monohydrate (Sigma-Aldrich M5885) glucose monohydrate (Sigma-Aldrich 49159 FLUKA) galactose monohydrate (Sigma-Aldrich G0750) or D-sorbitol (Sigma-Aldrich 240850) were filter-sterilized at the appropriate concentration and added to autoclaved nutrient supplements. 2% agar was used for single-cell measurements and for routine isolation of strains. Except for routine strain propagation, all carbon sources were filter-sterilized. YP was autoclaved and SC filter sterilized.

Standard protocols were followed for PCR-mediated transformation [1]. All transformations and mutant strain isolations used two rounds of streaking to single-cell-descendant colonies on YPD agar plates. In the case of the flocculent isolates that emerged in the evolution experiment, special care was taken to ensure isolation of a single clone. Very quickly (within a 1-minute time frame to diminish any toxicity of calcium / magnesium depletion) these were de-flocced in 0.1 M EDTA, centrifuged briefly (20s @ 6000 x g) and resuspended in buffer lacking calcium before streaking to singles. Later we found that these samples could be deflocced effectively in 20% mannose YP media and this media (2-3 ul containing appropriate cell count per 5 ml growth media) was used for inoculation into media for lag- and fluorescence measurements.

Strain S288c BY4741 (MATalpha) had to be specially engineered for this study. To enable maltose fermentation, it was transformed with MAL63 from sequence assembly contig 9 of RM11-1a to replace the coding region of MAL13[2], creating strain KV1156.

The second aspect that was critical for our study was to lower S288c’s petite frequency. We found that growing S288c logarithmically in glucose media for >20 hours leads to a frequency of petites that is on par with those reported by [3] (ranging from 40-80% of all cells). Cells that are petite, i.e. that have mitochondrial DNA mutations that lead to a lack of oxidative phosphorylation, are genetically unstable. Specifically, rapid nuclear genomic instability occurs due to the lack of oxidative phosphorylation and subsequent defective maturation of iron-sulfur clusters necessary to maintain genomic DNA integrity [4]. Because the critical glucose-growth phase of our evolution experiments required 20 hours of exponential growth, the generation of mutator phenotypes would definitely be a problem. We found that by rescuing S288c’s defective copy of SAL1 we could reduce the frequency of petites in these glucose-unlimited conditions to < 10% of colony forming units.

To generate the SAL1 allele, the strain RM11-1a was transformed with a PCR product generated by AN_GS1 and ANGS2 using the CaURA3 gene of pKT175 (Euroscarf) as template. This product integrated 193 bp downstream from the intact copy of SAL1. Genomic DNA from this strain was used as a template for a second PCR reaction using primers AN_GS3 and AN_GS4 that upon transformation into KV1156 rescued the SAL1 frameshift and simultaneously surrounded URA3 with a tandem repeat of the sequence downstram of SAL1. Subsequent selection for strains having URA3 popouts on 5-FOA medium yielded strain AN62, and these were checked by a PCR yielding products of appropriate size over the former URA3 locus. AN62 was transformed with the pSB283[1], treated with galactose to induce HO recombinase, and after curing of the plasmid by growth on 5-FOA, the subsequent MATa strain was named AN63. This strain was the starting strain for all subsequent experiments reported in this paper for S288c.

Constitutive over-expression of maltose permease and maltase (alleles MAL11 and MAL12 respectively) was achieved by transforming a GPD1 promoter directly upstream of the MAL gene coding sequences (primers AN_GS12 and AN_GS13 for MAL11 or AN_GS14 and AN_GS15 for MAL12) in a reaction with template pYM-N14; G418 selection; Euroscarf) to generate strains AN104 and AN105. The dual MAL11 and MAL12 over-expression strain AN107 was generated by transforming AN104 with the MAL12 over-expression construct (primers AN_GS10 and AN_GS11 in a reaction with template pYM-N15, nourseothrycin selection).

AN296 was generated by PCR-mediated transformation of AN63 with a THD3 promoter-driven YeCitrine construct published in Smukalla et. al. 2008 [5]. Primers KV1920 and KV1923 were used with strain KV1526 as genomic DNA template. This construct integrates near the YOR2 locus.

To generate AN148 we transformed AN63 first with a construct to label the MAL11 permease allele with YeCitrine using primers AN_GS6 and AN_GS7 with pKT174 as template (Euroscarf, KanR). The resulting strain AN77 was transformed to label the MalS allele MAL12 with a yeast codon-optimized mCherry (primers AN_GS8 and AN_GS9 using pSR101 [6].

AN74 was the reference strain used in the competitions reported in figure 3b and used as an “ancestral” control in figure 6b and S5c. This strain was made by transforming an mCherry allele fused with the MAL12 ORF using the same primers and mCherry template as those used to generate the AN148. AN73 was used in the maltase decay experiment reported in figure S2a. This was also made by transforming the MAL12 locus using the YeCitrine allele from pKT174 using the MAL12 primers. AN355, the MAL12-mCherry strain descended from isolate 1 was made in a similar fashion.

All genetic constructions involving (promoter or fluorescent protein) fusions to MAL11 and MAL12 were confirmed by checking the 5’ end of the construct with genome-specific primers AN_GS10 and AN_GS11 respectively and TEF promoter specific primer 69.

For specifics of the genetic complementation analysis of the HXK2 and STD1 mutations we identified by whole genome sequencing, please refer to the strain list. Generally, a KANMX marker was integrated downstream of the HXK2 or STD1 allele (in mutant and ancestral backgrounds), and this was used as a template for a PCR product that was used to transform the HXK2 or STD1 of the query strain (in ancestral to change to mutant or in the mutant to revert back to the ancestral allele). 16 independent transformants from each complementation set were confirmed to have the proper integration, then were screened in the bioscreen in 0.5% glucose, 2.5% galactose (LG + galactose). Samples matching the phenotype of interest were confirmed by sequencing by dye-terminator sequencing and glucose-to-maltose lags were measured under the microscope.

For the experiment reported in figure S5 and S8 where the MAL63 activator was deleted from Isolate 1, a PCR product was generated using primers AN177 and AN178 on the pUG6 template to generate strains AN443 and AN444. The PCR product was designed to integrate upstream of the hygromycin marker (which sits upstream of MAL63 in all the S288c strains reported in this manuscript) and downstream of the MAL63 activator coding sequence. This PCR product also shares homology with the terminator of the hygromycin marker, so the PCR product in some transformants integrated only in place of the hygromycin cassette. This PCR product was transformed into strain AN355 (Isolate 1 transformed with a MALS-mCherry cassette) and transformants selected on G418. Samples were patched to hygromycin-containing YPD plates to confirm that the transformation had resulted in deletion of the hygromycin cassette. Clones were then screened in maltose media to examine whether or not they could induce the MALS-mCherry reporter. Strains that could not activate their MALS gene in maltose-containing media were designated AN443, and strains that could still activate their MALS-mCherry reporter were designated AN445. Three independent transformants of each strain were used in the experiment and reported in figure S5 and S8.

*Media and general culturing methods for lag, fitness and gene expression measurements.*

All experiments with fluctuating environments (where cells were transferred from one medium to another) proceeded by centrifugation at room temperature at 1250 x g for 2-3 minutes. For centrifugation of cell populations growing in SC media at cellular densities less than 1*10^6 / ml, we found we needed to add 10% YP in order to form a visible cell pellet. Samples were always resuspended in SC media after the final wash.

For samples with longer than 8 hours of logarithmic growth in glucose, cultures were diluted into 2-5 ml media in plastic disposable falcon or round-bottomed tubes containing the appropriate media. For these samples, initial OD600 was determined and cultures were diluted such that they would be between 1-5*10^6 cells/ml at time of harvest for transfer to a new environment.

*Diauxic shift measurements using Bioscreen C*

All diauxic shift growth measurements reported in this manuscript were made from one batch of media containing 1% glucose dissolved in 2x YP. An equal volume of appropriately concentrated sugar supplements was mixed with this batch of media to make the final sugar + 0.5% glucose + YP media. Media was kept at room temperature for no longer than 1 week and otherwise kept frozen in a -20 C freezer.

We found that important parameters – in particular lag phases and thus GMR – were profoundly affected by the presence of fermenting cells in neighboring wells. To control for these unwanted artifacts, samples were incubated in the 100-well honeycomb Bioscreen plates with at least 5 wells containing media surrounding each sample (descending sides of plate were never used) and therefore never with another fermenting sample adjacent. Therefore, in a 1-plate, 100-well bioscreen experiment the maximum number of independently growing cultures we used was 20 wells. This technical aspect, in addition to only using one batch of media (kept in the freezer until day of use) was critical to obtain reproducible results on different days.

Experiments screening mutants and wild strains were performed with at least two biological replicates, and for controls e.g. ancestral strains, 4 biological replicates. Samples that behaved inconsistently between biological replicates were repeated again, this time with three or four biological replicates. Single colonies were isolated by streaking from glycerol stocks in the -80 C freezer on 2% agar plates containing solid YP + 2% glucose (YPD) media. Colonies were inoculated in 96-well plates (Corning 3959) in 150 ul of medium containing YP and 2% glucose. After mixing, 2 ul of sample was diluted into 150 ul of medium and the plate was sealed and set on a 1.5 mm orbital shaker at 1000 RPM overnight. After 18-24 hours growth, this turbid culture was diluted 3-fold to a total volume of 150µl and OD600 measurements were made with a plate reader (Versamax, MD Biosciences). To make equal initial inoculates, 10*1/OD600 µl of these diluted cultures were then transferred to a total volume of 150 µl YP, and 3 µl of this culture was then used to inoculate 147 ul of the growth medium for approximately a 5,000-fold total dilution of turbid overnight culture. Growth curves were obtained for 2-3 days, until most cultures in a plate had reached an OD600 of 1.15.

*Bioscreen C growth curves for data presented in figure S8a.*

Because of time and space constraints (in order to be able to measure 3 biological replicates * 36 mutants + WT in conditions coming from glucose and maltose all in one experiment), we had to disregard the effect that neighboring Bioscreen wells with fermenting cells plays in affecting growth properties. Samples were inoculated from mid-logarithmic phase growth in YP media containing 20% maltose or 10% glucose optical densities such that maximal growth rates were detectable 7-8 hours after inoculation.

*Analysis of Bioscreen C growth curve data*

R and Excel software programs were used for all data manipulation and statistical analyses. To analyze growth curves from the Bioscreen C, we first applied R’s *smooth.spline* function to the raw data with respect to time. For analysis of growth rate data, we found that setting a *spar* value of 0.35 was necessary to remove measurement noise caused by variable measurements of the Bioscreen, especially at background-subtracted OD600 values ≤ 0.25, where we found the coefficient of variation between consecutive measurements of non-growing cultures to be highest. To represent instantaneous growth rates as a function of population size and compare these across samples, it was necessary to interpolate missing values of growth rates and OD600. To do this we first encoded log-transformed smoothed OD600 data as a function of time using *splinefun*. Finding the first derivative gave the instantaneous growth rate as a function of time, and then a new function of the first derivative as a function of OD600 values was made using *splinefun,* effectively giving the specific growth rate as function of population size. An output file was generated interpolating growth rates across a sequence of 100 OD600 values from 0.15 to 0.75. This was necessary to allow for visual comparisons across many samples and to allow reproducible GMR calculations (see below and the Dataset S6).

The script smoothens data and interpolates growth rates. Such analysis can be performed by hand by simply making an XY scatterplot of growth rates as a function of the measured population size for this particular sample, and therefore each sample has a separate set of XY coordinates. By contrast, the script interpolates growth rates across defined population sizes, allowing one set of horizontal coordinates that is applicable to all samples. The methods are comparable, however the script removes noise and allows comparison across many strains at exactly the same OD600 values.

Maximum growth rates are the average of the three highest interpolated growth rate measurements. To calculate GMR, we determined the amount of time t that the culture spent between OD600 between 0.15 and 0.0.75, where time t for each i'th OD600 equals (lnODi-lnOD(i-1))/r , with r equal to the instantaneous growth rate at that point. This approach reduces error (the coefficient of variation between biological duplicates) significantly compared to the simpler approach which would only use the raw data from the bioscreen. This is because GMR calculations based upon raw data are discrete in the time dimension and indiscrete in the OD600 dimension. Thus, if the culture reaches an OD600 value very close to 0.15 or 0.75, the GMR calculation will very accurately reflect the true GMR. Alternatively, if the OD600 values are relatively far away from the OD600 values 0.15 or 0.75, the calculation will be inaccurate. Therefore, provided that growth rates are greater than zero during the course of the experiment (which will lead to a mis-estimation of the duration of the lag phase), the interpolated OD600 values give a more reliable GMR measurement across samples. No samples reached a growth rate of zero except one culture of YS4, reported in the first two panels of figure 1.

*Construction of chambers for single-cell lag and doubling time measurements.*

To measure single-cell lags and doubling times of many samples at once we devised a system that presses cells between an agar pad containing media necessary for growth and a coverslip for recording using an inverted microscope. To make the chambers, we fixed a coverslip to the bottom of a plastic plate that would fit in the microscope, and after we would pour agar media on top. To do this we used a red-hot scalpel to cut a rectangular hole in a polystyrene plate (Nunc part no. 242811) of lesser dimensions than a 24 x 50 mm #1 glass coverslip. Using another sharper scalpel, we whittled away the melted plastic bits on the edges of the hole to allow the coverslip to sit evenly. Then we used household acetic acid-soluble silicone sealant (such as that used in making fish tanks or sealing bathroom tiles) to affix the coverslip to the bottom of the plate, scraping away the sealant that spread out from between the coverslip and plate to allow microscope objective to move around the entire area of the coverslip. After at least 2 days’ time to allow for curing of the silicon sealant, we sprayed the inside of the plate with distilled water and 70% ethanol to wash away dust and contaminants. We have never found further sterilization of the chambers to be necessary.

*Details of sample preparation for microscopy.*

We found that careful treatment of samples prior to transfer to the growth chamber was extremely important to make reproducible results, and so we discuss in detail here the various technical aspects of sample preparation for the microscope. Media for microscopic analysis always contained 2% agar and either 1x YP (all single-cell lag measurements except where fluorescence microscopy was used) or 1x SC (lag measurements in figure 2 and microscopic growth rate measurements in figures 2 and S8c). SC media was boiled 2-3 times in the microwave to sanitize and melt agar, and YP media was autoclaved for 20 minutes at 2 X concentration, and brought to the proper concentration of sugar + media after autoclaving. 90 ml of liquid 40-60 degrees C 2% agar containing the necessary nutrient supplement was poured into the modified Nunc plates and allowed to set at room temperature for at least an hour.

Once samples were ready for analysis, the solidified agar pad was popped out, bottom-side up, using a 200 ul pipette tip run along the periphery between the agar and the sides of the plate, onto a piece of cling plastic wrap. Because of the hole cut in the bottom of the plate for setting the coverslip, the agar has a protruding plateau corresponding to the media that set against the coverslip. 1.5-2.5 ul of prepared sample corresponding to 1000-5000 cells was then spotted onto the agar pad plateau and allowed to dry for 10-25 minutes in a sterile laminar flow hood. Up to 30 spots can be deposited using a coverslip of these dimensions. The modified Nunc plate was then put back on top of the agar pad and the agar pad pressed to remove air bubbles and to ensure the spotted cells were pressed up against the coverslip. The plate was sealed with the plastic wrap remaining against the top of the agar pad, with the lid fitted such that excess plastic wrap was wedged between the plate and lid to make an acceptable seal. We found that shifting of samples presumably due to evaporation was rarely a problem. The modified plate + agar + media + cells was placed in the inverted TiE microscope chamber, which had been acclimated to 30 degrees for at least 4 hours. Due to the warming up process, the modified plate appears to move about 100-150 microns downwards toward the objective within the first 2 hours of movie acquisition. To accommodate this, we set “redefine Z position after focus” on the microscope and sometimes if cells had very long lag phases, we would just wait for 1-2 hours for the growth chambers to acclimate.

*Details of experimental procedures for single-cell lag measurements.*

Single-cell lag measurements were performed with growth in 10% glucose YP media and lag measurements took place in 20% maltose YP-containing media. We chose 10% glucose for these conditions because during the experimental evolution protocol some samples began to grow faster and deplete glucose; thus to maintain “high glucose” conditions we compensated with higher concentrations of glucose. We found in preliminary experiments that similar results are achieved for lower concentrations of glucose and maltose as long as osmolarity between the conditions is kept constant. Samples were washed by 1250xg centrifugation two times with 20% maltose. After washing, samples were then resuspended in 20% maltose such that cell densities were about 0.5-1*10^7 cells / ml.

*Details of microscopy.*

All microscopy was performed on a Nikon TiE automated microscope equipped with a Nikon Plan Apo VC 60x, 1.40 NA oil objective encased in temperature-controlled incubator set to 30ºC. An Andor LucaR 604 camera was used for all microscopy. A dichroic Semrock YFP-2427B-000 filter (500/24 nm exciter and 542/27 nm emitter) was used for all YFP measurements, and for mCherry measurements a Semrock mCherry-B-000 (562/40 exciter and 641/75nm emitter). Epifluorescent light came from a Sutter Lamda XL lamp. To maximize sensitivity and minimize photobleaching in fluorescence microscopy experiments, 8x8 camera binning was used with 25% lamp intensity and narrow aperature settings. Exact camera exposure times were determined before beginning of each experiment on samples that were not included in final analysis.

The microscope allows monitoring of many different XY positions in one experiment. Within a spot of cells on the agar pad in the growth chamber, we would pick between 5-10 xy positions in order to make time-lapse movies; in the end with 3 spots minimum and 24 maximum per experiment, 30-120 positions were picked. Images were acquired every 15-20 minutes. For growth rate measurements, images were acquired every 5 to 6 minutes for up to 70 positions.

*Analysis of time lapse movies for representation of single-cell lag and doubling time measurements.*

Cell budding events were scored by hand as the hour at which the first morphological change leading to a new bud occurred, or alternatively when an already existing bud began to grow. We found that cell cycle status – i.e. whether or not cells had buds – was not a meaningful predictor of lag phase escape and so disregarded this information for analysis. For doubling time measurements presented in figure 2, the time between the beginning of the first bud to the beginning of the third bud was recorded (equivalent to the average of two generations). For mother cells already in the process of budding, the time to the equivalent cell cycle phase was recorded. For mother cells in the late stages of the process of budding, we waited for the maturation of the daughter cell to complete, with the emergence of the next bud recorded as the first time point.

*Microcolony growth rates are reported in figure S8.*

Due to cell cycle variation between mother and daughter cells, microcolony growth rates are stochastic and noisily distributed for small population sizes (1-3 cells) due to asymmetric cell division characteristic of budding yeast [7]. Therefore, we measured all microcolonies of starting cell sizes > 8 cells, corresponding to 3 hours into the recording of the movie. Using NIS elements software, we drew a ROI (using NIS Elements software, Nikon) around an initial colony, then a ROI of that same colony 3 hours later. ROI information included the area of the microcolony as well as the fluorescent signal. The ln(area_final/area_init)/time was taken to be the specific growth rate or the colony at that time point.

For fluorescence microscopy analysis, at each position, three or four rectangular ROIs were drawn around regions with no cells. The average of these ROIs was used to subtract background signal from single-cell or microcolony measurements.

*Selection experimental protocol.*

For each evolution experiment, the paradigm established by [8] was followed. All growth was in YP media at 30 degrees C on a rotating wheel. The protocol was followed for two founding S288c strains: AN296 (constitutively mCitrine labeled) and AN148 (MAL-genes labeled). For each experiment, we inoculated one 2-day old colony resurrected from the freezer stock of the strain growing on a YPD plate directly into 3 ml of 20% maltose and grew it for 24 hours. 1 ml of this culture was stored at -80 C in 25% glycerol as the “ancestral” progenitor strain. With the remaining culture, we diluted the population down to an estimated 6-8000 cells/ml in 5 ml of 10% glucose YP across 12 15 ml conical bottomed plastic tubes (Greiner). After 20-22 hours growth on the wheel (population densities of 0.5-1*10^7 cells/ml), we centrifuged the populations at 1250 x g for 5 minutes, resuspended in 5 ml 20% maltose YP, vigorously resuspended the cultures by shaking, spun again, and then resuspended again in 5% ml 20% maltose media by vigorous shaking. Samples were then put back on the wheel for another 3 days until the populations reached densities of 5*10^8 cells / ml. After each round of selection in maltose, we froze an aliquot in 25% glycerol at -80 for future analysis and resurrection.

For AN148, during rounds 1, 3, 5, 7 and after the completion of round 8, we measured single-cell lag phases at the end of the glucose leg of the selection cycle in the microscope as described above.

*Malthusian fitness competitions*

All competitions were performed with labeled reference cells (AN74) harvested from one large flask, split into the various media/environments where the competitions would occur between different biological replicates of query strains (e.g. WT or ancestral, or galactose or maltose-adapted cultures). Samples would be mixed at high culture densities for measurement of optical densities and diluted appropriately for the experiment at hand.

For the experiment reported in figure 3b, we pre-grew query (isolates 1, 3, and 4) in sextuplicate and mixed these 1:1 with one large batch of AN74 reference cells exactly as in the enrichment protocol in 20% maltose YP media for 24 hours. OD600’s were determined and query cultures were mixed at a 1:1 ratio between reference and query strains. Samples were frozen for initial ratio measurements. Samples were then diluted to a density of ~8000 cells/ml in 5 ml of 10% glucose YP, diluted on YPD agar plates for single-colonies to estimate the initial population size, and left the remainder of the culture to grow for 20 hours. Samples were centrifuged, washed 2 times with 20% maltose YP, and then resuspended in 5 ml 20% maltose and allowed to grow for the next 48 hours. Samples were removed from the competition environment, frozen in 25% glycerol for later analysis, and final population densities determined by plating to singles. For flow cytometry analysis, see below.

*Analysis of competitions*

Assuming that the reference strain would behave the same regardless of which query strain it was competing against, we calculated the Malthusian growth rate of the query

$$w_{query}=\ln\left( \frac{{fraction}_{query final}\cdot{population size}_{final}}{{fraction}_{query initial}\cdot{population size}_{initial}} \right)$$

Likewise w(reference) was calculated for the fluorescently labeled reference. The w(query)/w(reference) was taken as the fitness of the query strain. This value divided by the ancestral strain’s fitness (calculated identically against the same reference) gives the fitness of the query strain. All fitness or relative growth rate measurements are the ratio of the query strain’s fitness relative to the reference strain divided by the control query strain’s fitness relative to the reference strain. Error bars represent standard deviation and account for error of both the query and control:

$$\sigma_{r}=\frac{1}{r}\sqrt{\left( \frac{\sigma_{w_{query}}}{w_{query}} \right)^{2}+\left( \frac{\sigma_{w_{ancestral}}}{w_{ancestral}} \right)^{2}}$$

*Flow cytometry analysis*

50,000 single-cell events were acquired by a BD Biosciences Influx flow cytometer. mCherry signal detection used a 561 nm laser coupled to a 610/20 nm detector and YeCitrine signal detection used a 488 nm laser coupled to a 580/30 nm detector.

For analysis of competitions, there were always clearly delineated groups of mCherry-labeled cells (representing the reference strain AN74) and mCitrine-labeled cells (representing the query strain, either the ancestral AN296 or its short-lagged descendants). < 0.1% of events were fluorescent in both channels; these likely autofluorescent dead cells were excluded from analysis. Gates were drawn around mCherry and mCitrine events, and total count for the query strain divided by the sum of both groups was taken to represent the fraction of the population represented by the query strain, important for future analyses of competitive fitness (see below).

For analysis of gene expression, after list acquisition, SSC and FSC outliers -- about 20 to 30% of events -- were filtered out in FlowJo software and the rest of the events exported to a spreadsheet where total fluorescence per cell was calculated or histograms made. For histogram traces in figure 6 they were binned 10x. Traces in figure 6b were made using standard features of FlowJo software.

*Whole genome sequencing and variant calling*

Selected samples were whole-genome sequenced using Illumina HiSeq 2000 with 500bp inserted library. Quality assessment of resulted short reads was performed using FASTX-Toolkit (http://hannonlab.cshl.edu/fastx_toolkit/index.html). After removing the low quality reads (below Q30) and adaptors, pair-end reads were then mapped onto the reference *S*. *cerevisiae* genome (S288C, version genebank64) using Burrows–Wheeler Alignment [9]. Default settings were used except the maximum edit distance was set to 0.01 (-n 0.01). The MarkDuplicates command in Picard (http://picard.sourceforge.net/) was used to remove the reads that mapped to the same positions in the reference genome (PCR duplications). Consensus Single-nucleotide variations (SNPs) and small insertions and deletions (Indels) were called for each chromosome using SAMtools and GATK [10,11]. Default settings were used except the maximum read depth in SAMtools was set to 150X (-D 150). The generated SNPs and Indels were then filtered to minimize the false positive mutation calls. First, SNPs and Indels lying in low complexity sequences (such as telomeric, subtelomeric, transposon, repeat regions and et al…) were filtered out. Second, mutations with a total read depth below 20X were discarded. Third, SNPs and Indels with a quality score below 30 were removed. Forth, mutation calls were only kept when at least 80% of the reads were positive for the SNP sites. Only the SNPs/Indels which were verified by both GATK and SAMtools were kept as confident sites. The lists of SNPs/Indels were then annotated by in-house Perl scripts with the yeast genome database [12]. CNV-seq [13] was used to identify consecutive regions along the chromosome that show abnormal log2-ratios which indicated the potential copy number variation (CNV). Only regions larger that 1Kb were considered as CNV region.

*Supplemental references*

1. Weissman J, Guthrie C, Fink GR (2010) Guide to Yeast Genetics. Academic Press. 1 pp.

2. Brown CA, Murray AW, Verstrepen KJ (2010) Rapid Expansion and Functional Divergence of Subtelomeric Gene Families in Yeasts. Current Biology 20: 895–903. doi:10.1016/j.cub.2010.04.027.

3. Dimitrov LN, Brem RB, Kruglyak L, Gottschling DE (2009) Polymorphisms in Multiple Genes Contribute to the Spontaneous Mitochondrial Genome Instability of Saccharomyces cerevisiae S288C Strains. Genetics 183: 365–383. doi:10.1534/genetics.109.104497.

4. Veatch JR, McMurray MA, Nelson ZW, Gottschling DE (2009) Mitochondrial Dysfunction Leads to Nuclear Genome Instability via an Iron-Sulfur Cluster Defect. Cell 137: 1247–1258. doi:10.1016/j.cell.2009.04.014.

5. Smukalla S, Caldara M, Pochet N, Beauvais A, Guadagnini S, et al. (2008) FLO1 Is a Variable Green Beard Gene that Drives Biofilm-like Cooperation in Budding Yeast. Cell 135: 726–737. doi:10.1016/j.cell.2008.09.037.

6. McClean MN, Hersen P, Ramanathan S (2011) Measuring in vivo signaling kinetics in a mitogen-activated kinase pathway using dynamic input stimulation. Methods Mol Biol 734: 101–119. doi:10.1007/978-1-61779-086-7_6.

7. Hartwell LH, Unger MW (1977) Unequal division in Saccharomyces cerevisiae and its implications for the control of cell division. J Cell Biol 75: 422–435.

8. Elena SF, Lenski RE (2003) Microbial genetics: Evolution experiments with microorganisms: the dynamics and genetic bases of adaptation. Nat Rev Genet 4: 457–469. doi:10.1038/nrg1088.

9. Li H, Durbin R (2009) Fast and accurate short read alignment with Burrows-Wheeler transform. Bioinformatics 25: 1754–1760. doi:10.1093/bioinformatics/btp324.

10. Li H, Handsaker B, Wysoker A, Fennell T, Ruan J (2009) The sequence alignment/map format and SAMtools. Bioinformatics 25:2078-9. doi: 10.1093/bioinformatics/btp352.

11. McKenna A, Hanna M, Banks E, Sivachenko A (2010) The Genome Analysis Toolkit: a MapReduce framework for analyzing next-generation DNA sequencing data. Genome Res. 20: 1297-1303. doi:10.1101/gr.107524.110

12. Cherry JM, Adler C, Ball C, Chervitz SA (1998) SGD: Saccharomyces genome database. Nucleic acids Res. 26: 73–79.

13. Xie C, Tammi MT (2009) CNV-seq, a new method to detect copy number variation using high-throughput sequencing. BMC Bioinformatics 10:80. doi:10.1186/1471-2105-10-80
